# Supplementary material for: The asymmetric expression of HSPA2 in blastomeres governs the first embryonic cell-fate decision
Source: eLife. 2025 Mar 10;13:RP100730. doi: 10.7554/eLife.100730 (PMC11893103; doi:10.7554/eLife.100730)
Supplement: Figure 1—source data 1. [file elife-100730-fig1-data1.zip › Figure 1-Source data 1.pdf]

HSPA2

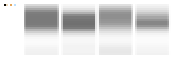

ACTIN

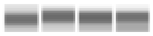

Figure 1, Source Data 1. Original membranes corresponding to Figure 1, panel F.

Figure 1, Hspa2 is asymmetrically expressed between 4-cell blastomeres. F. 4-cell embryos were separated into individual four blastomeres which were then analyzed by Jess TM Simple Western System based automated western immunoblotting with high sensitivity.

HSPA2

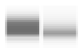

ACTIN

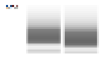

Figure 1, Source Data 1. Original membranes corresponding to Figure 1, panel I.

Figure 1, Hspa2 is asymmetrically expressed between late 2-cell blastomeres. I. Late 2-cell embryos were separated into individual two blastomeres which were then analyzed by Jess TM Simple Western System based automated western immunoblotting with high sensitivity.
