# Supplementary figures and images for: The asymmetric expression of HSPA2 in blastomeres governs the first embryonic cell-fate decision
Source: eLife. 2025 Mar 10;13:RP100730. doi: 10.7554/eLife.100730 (PMC11893103; doi:10.7554/eLife.100730)

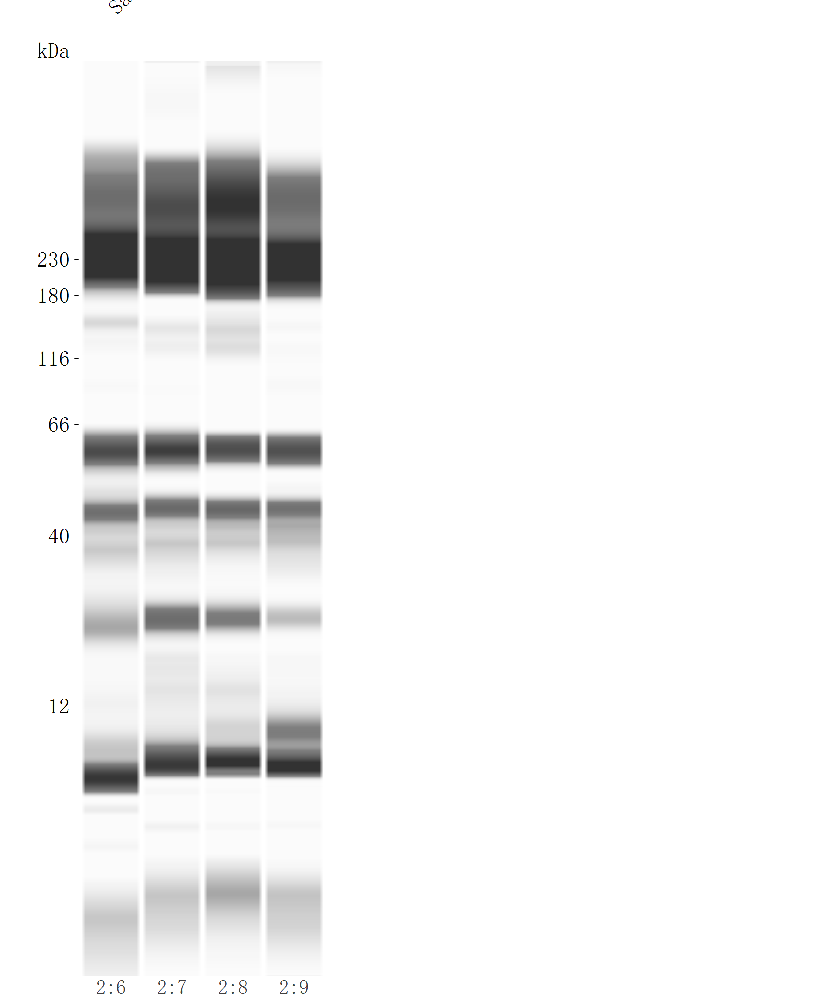

Supplement: Figure 1—source data 2. [file elife-100730-fig1-data2.zip › Figure 1F ACTIN.png]

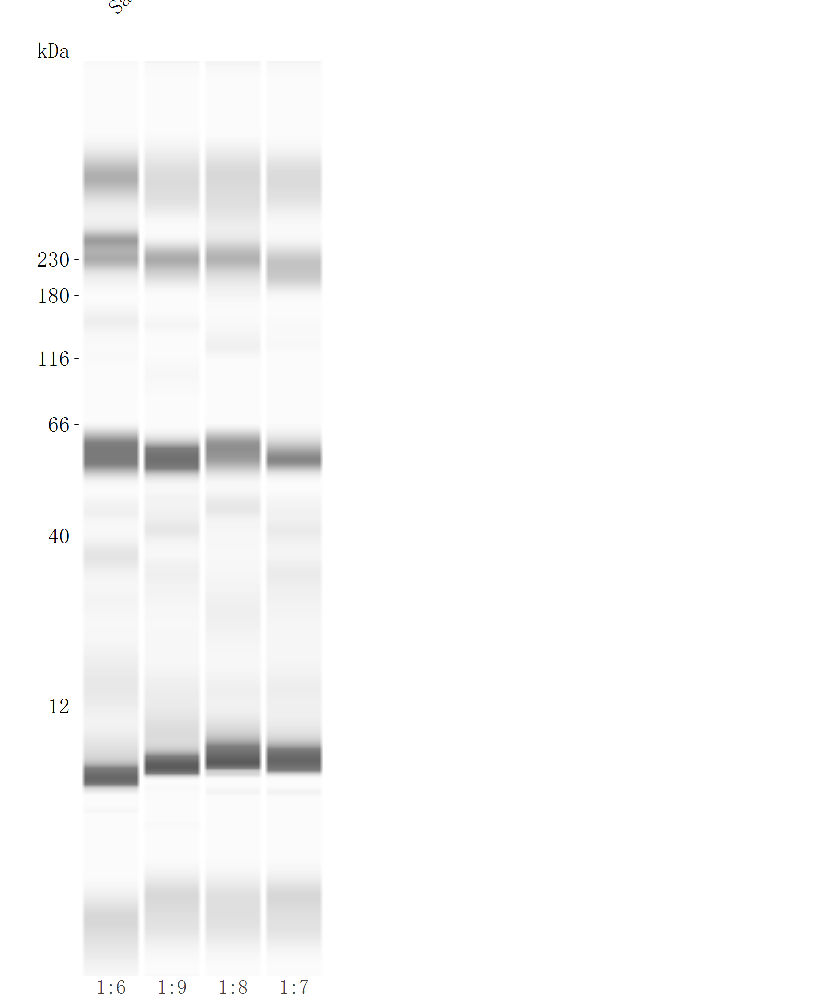

Supplement: Figure 1—source data 2. [file elife-100730-fig1-data2.zip › Figure 1F HSPA2.png]

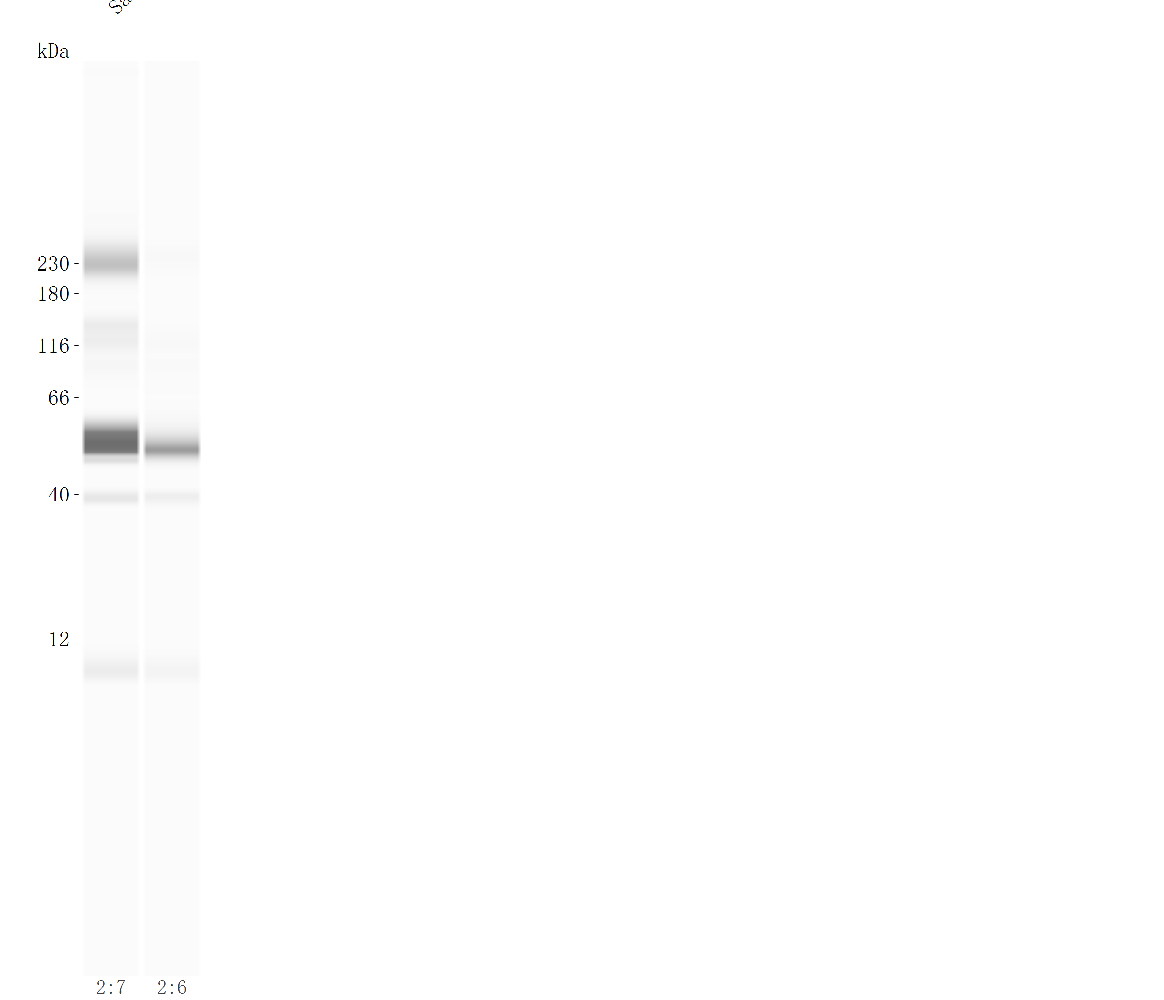

Supplement: Figure 1—source data 2. [file elife-100730-fig1-data2.zip › Figure 1I HSPA2.png]

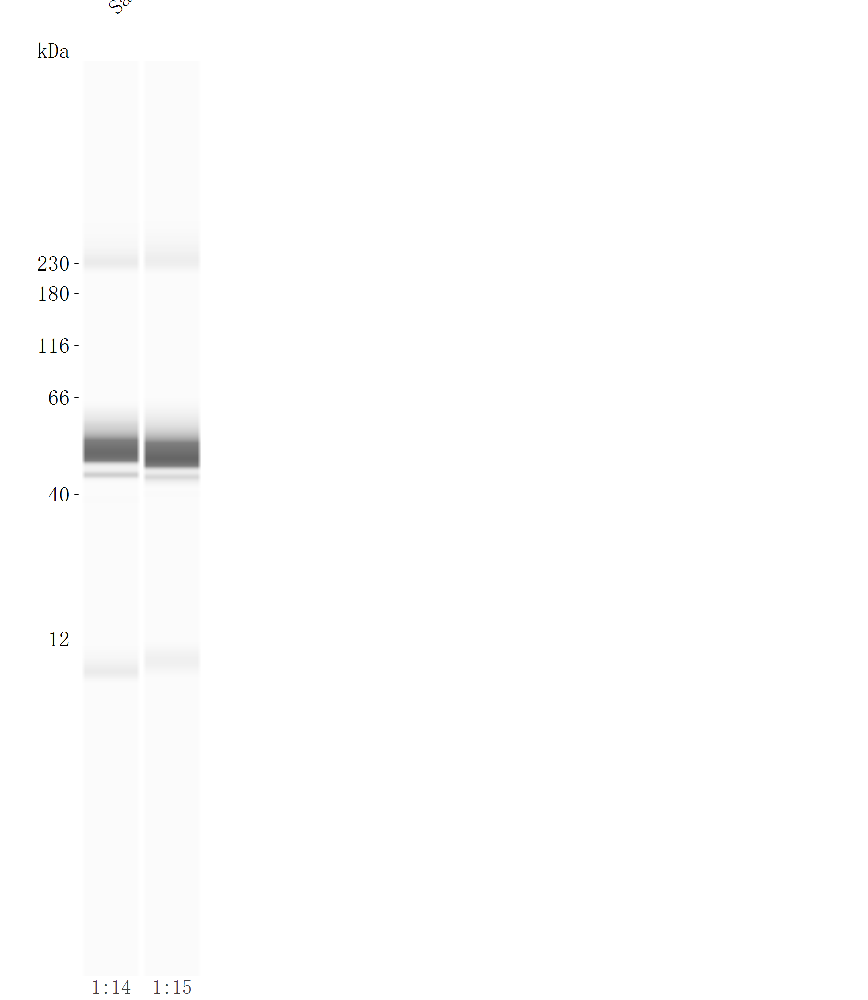

Supplement: Figure 1—source data 2. [file elife-100730-fig1-data2.zip › Figure 1I ACTIN.png]

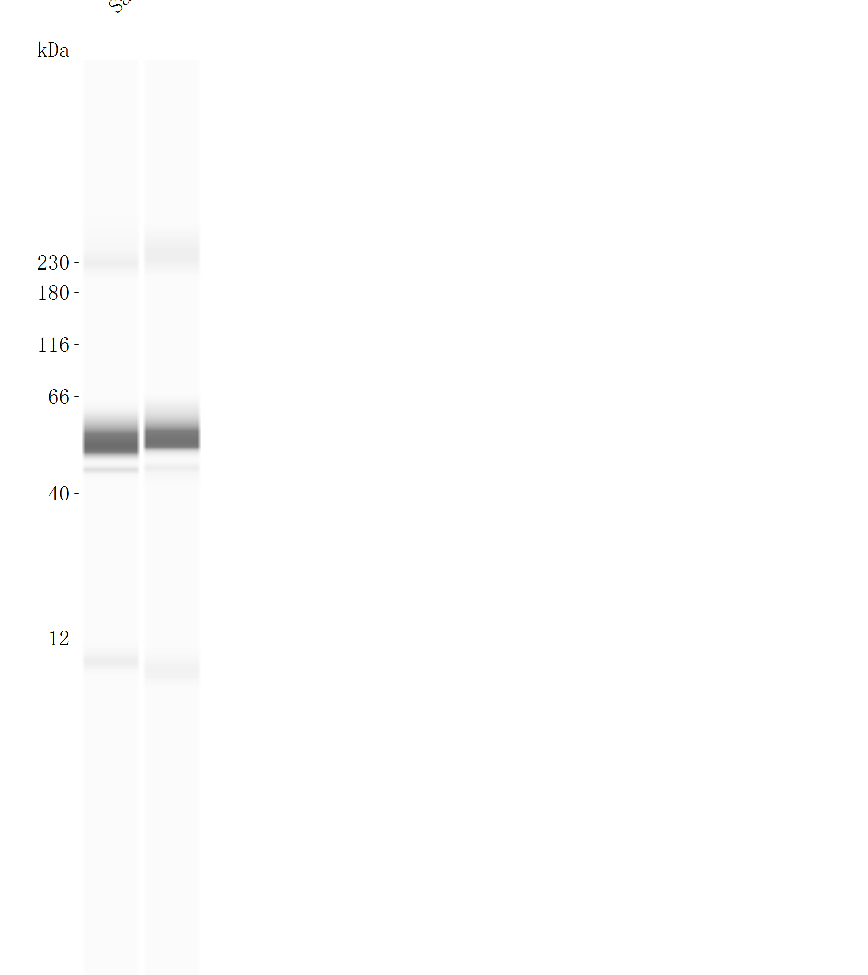

Supplement: Figure 1—figure supplement 1—source data 2. [file elife-100730-fig1-figsupp1-data2.zip › ACTIN.png]

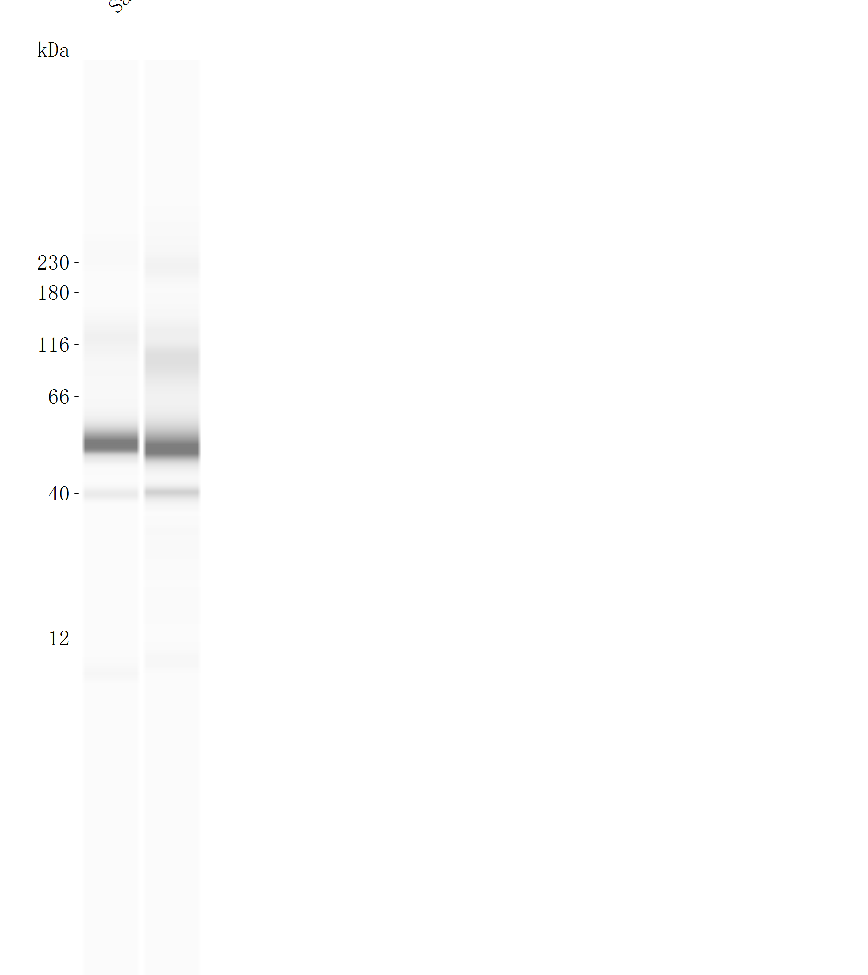

Supplement: Figure 1—figure supplement 1—source data 2. [file elife-100730-fig1-figsupp1-data2.zip › HSPA2.png]

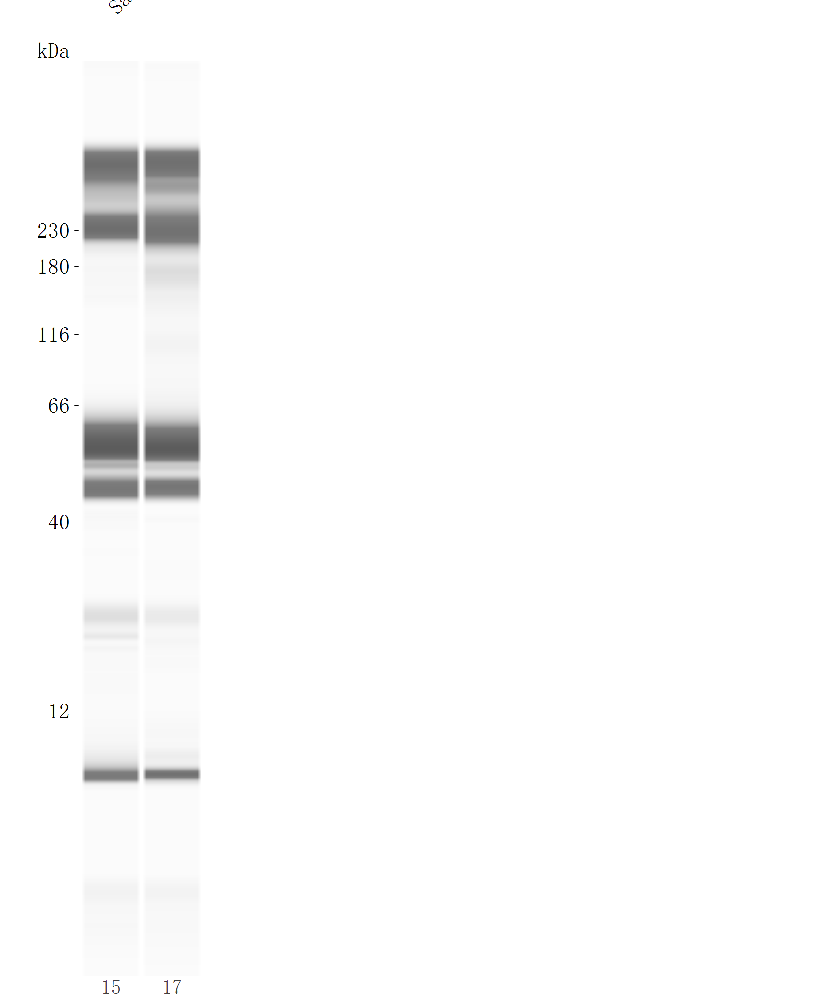

Supplement: Figure 2—source data 2. [file elife-100730-fig2-data2.zip › Figure 2K CDX2.png]

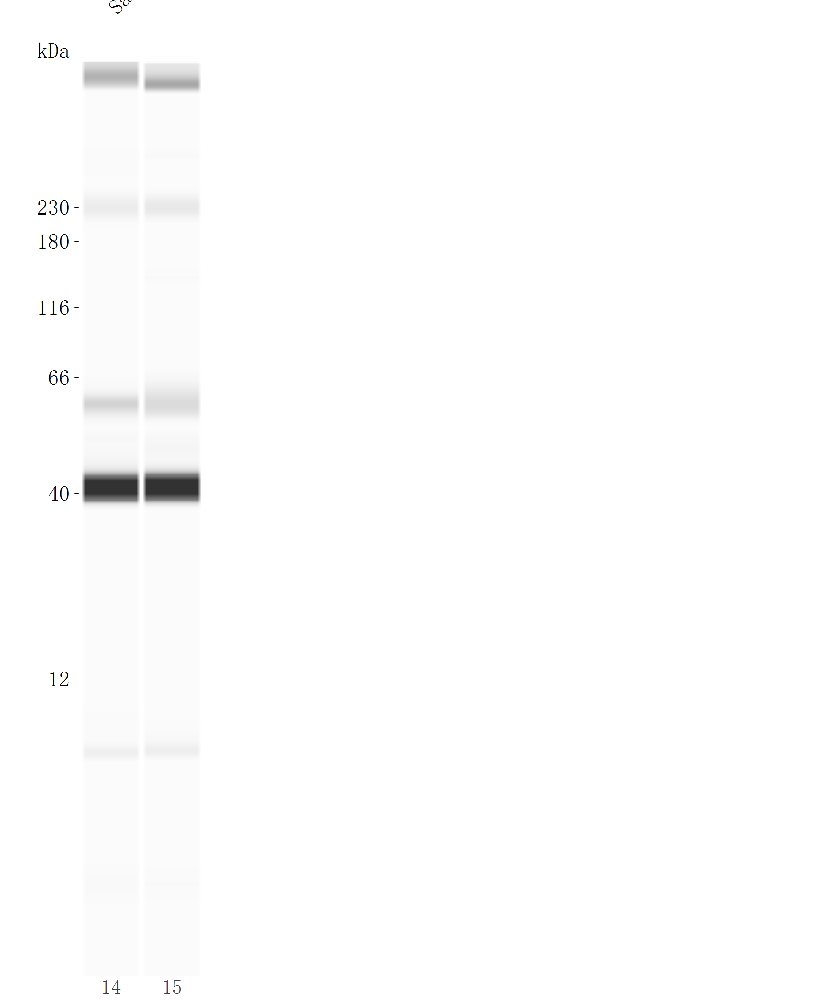

Supplement: Figure 2—source data 2. [file elife-100730-fig2-data2.zip › Figure 2K GAPDH.png]

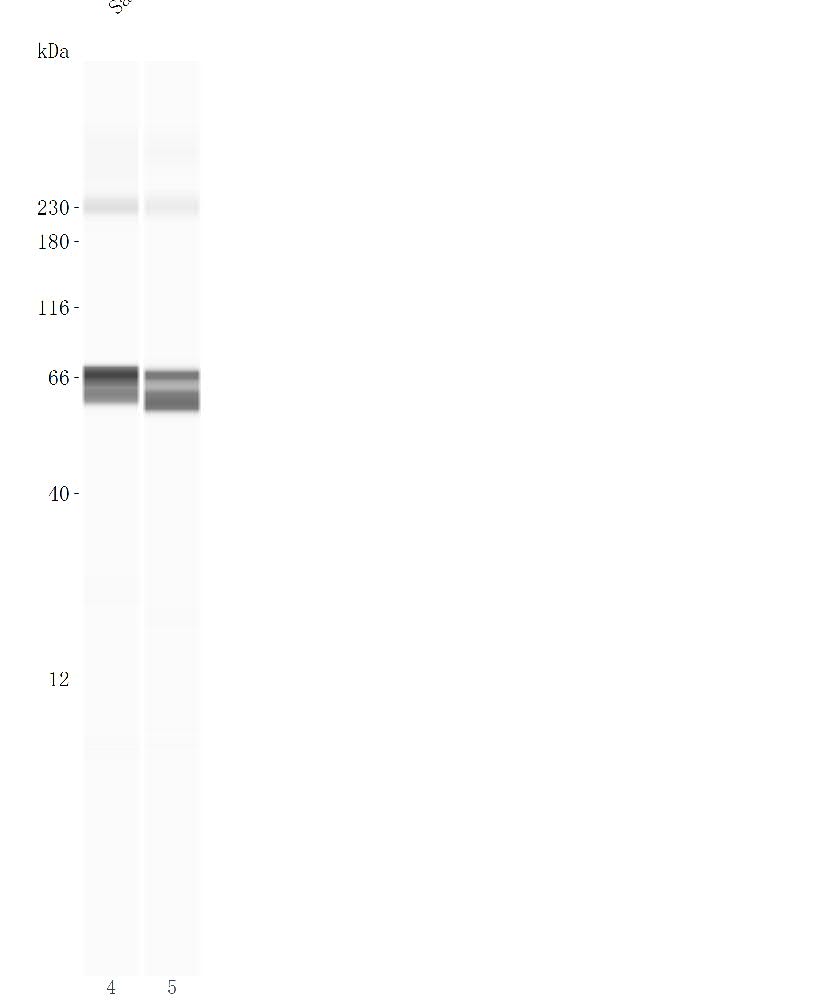

Supplement: Figure 2—source data 2. [file elife-100730-fig2-data2.zip › Figure 2K HSPA2.jpg]

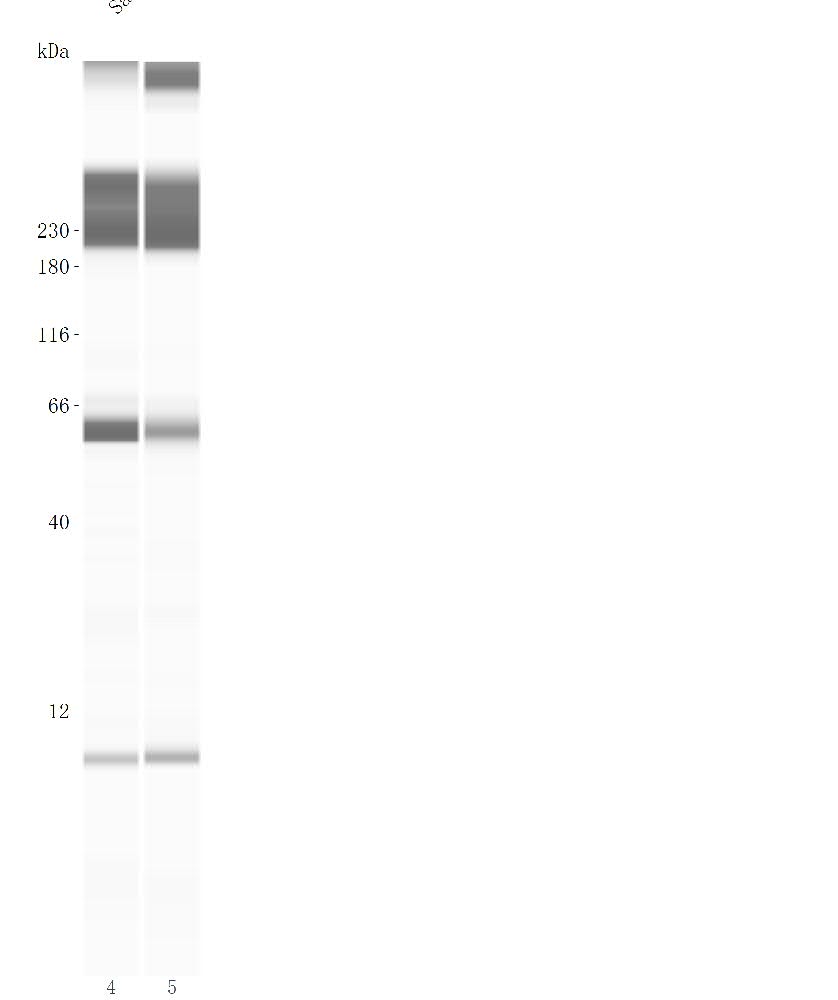

Supplement: Figure 2—source data 2. [file elife-100730-fig2-data2.zip › Figure 2K OCT4.jpg]

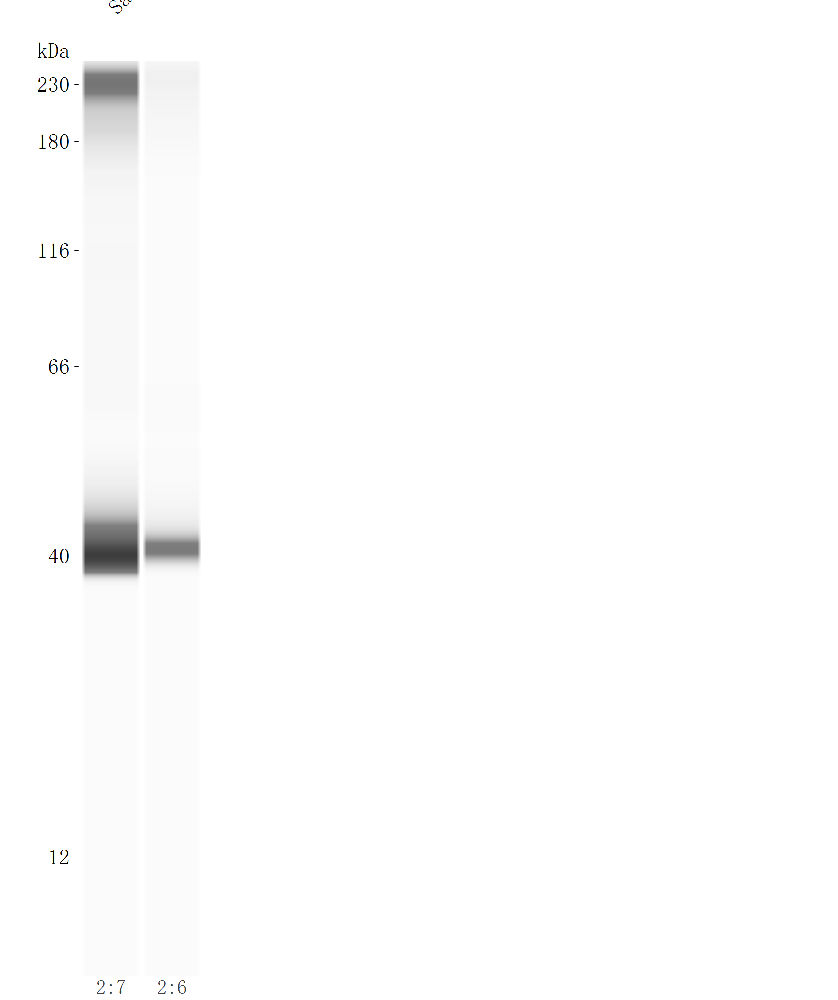

Supplement: Figure 2—source data 2. [file elife-100730-fig2-data2.zip › Figure 2K SOX2.png]

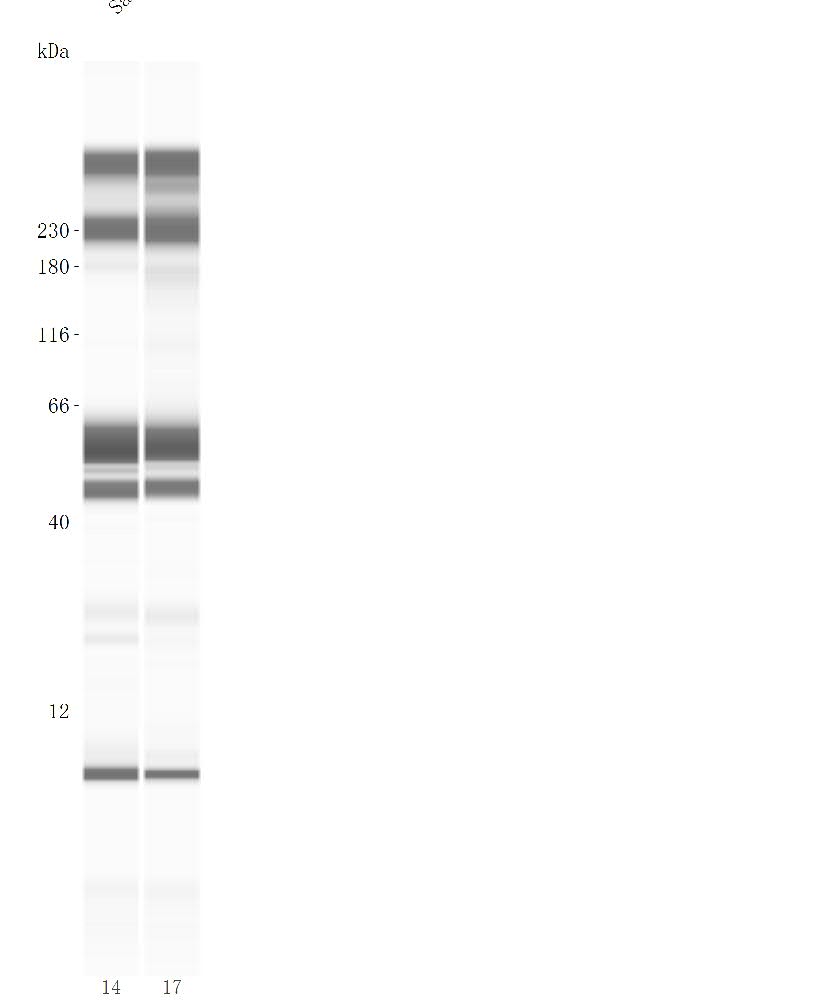

Supplement: Figure 2—source data 2. [file elife-100730-fig2-data2.zip › Figure 2L CDX2.jpg]

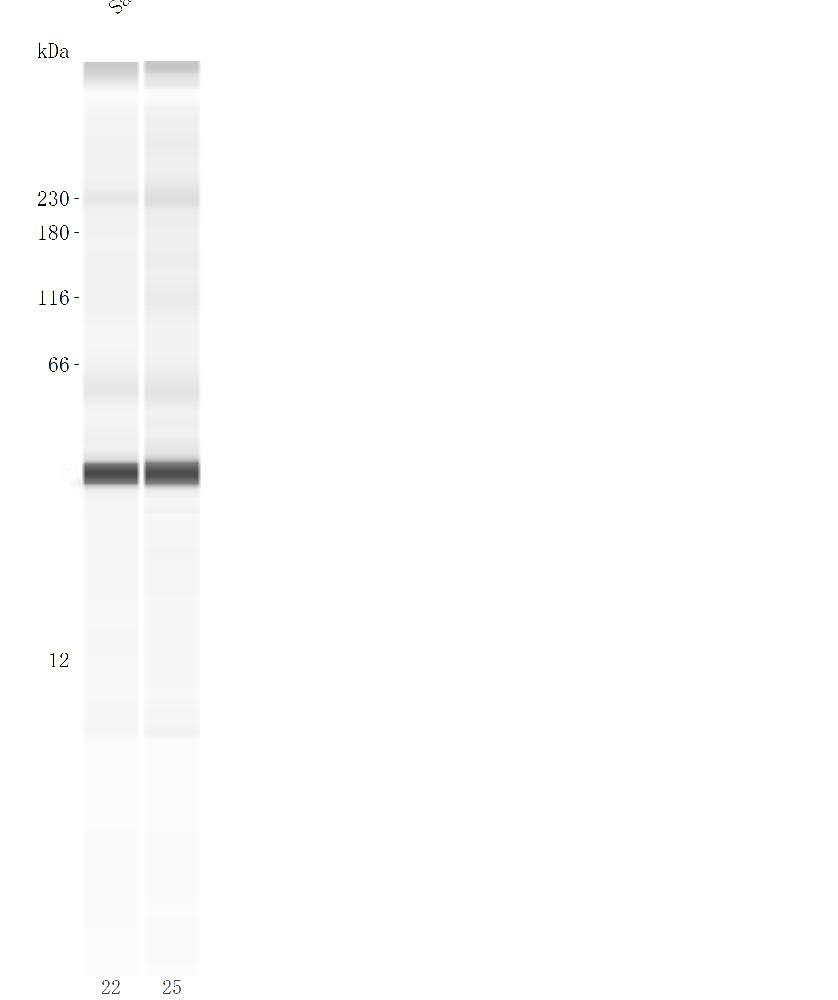

Supplement: Figure 2—source data 2. [file elife-100730-fig2-data2.zip › Figure 2L GAPDH.jpg]

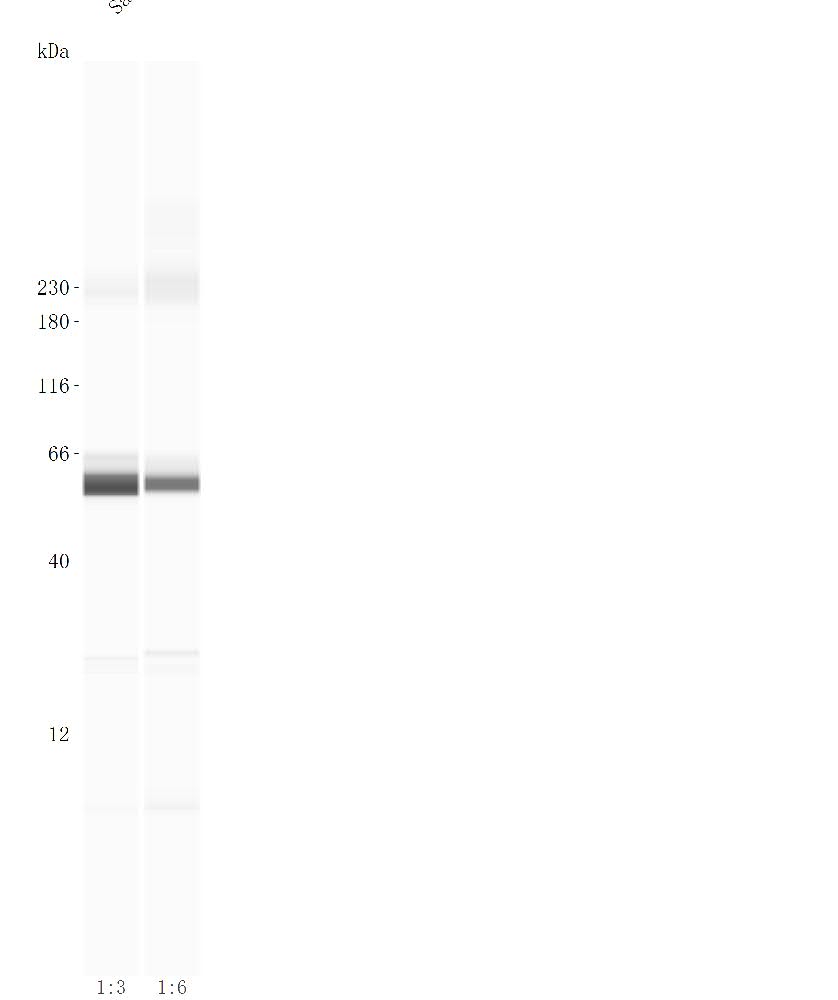

Supplement: Figure 2—source data 2. [file elife-100730-fig2-data2.zip › Figure 2L HSPA2.jpg]

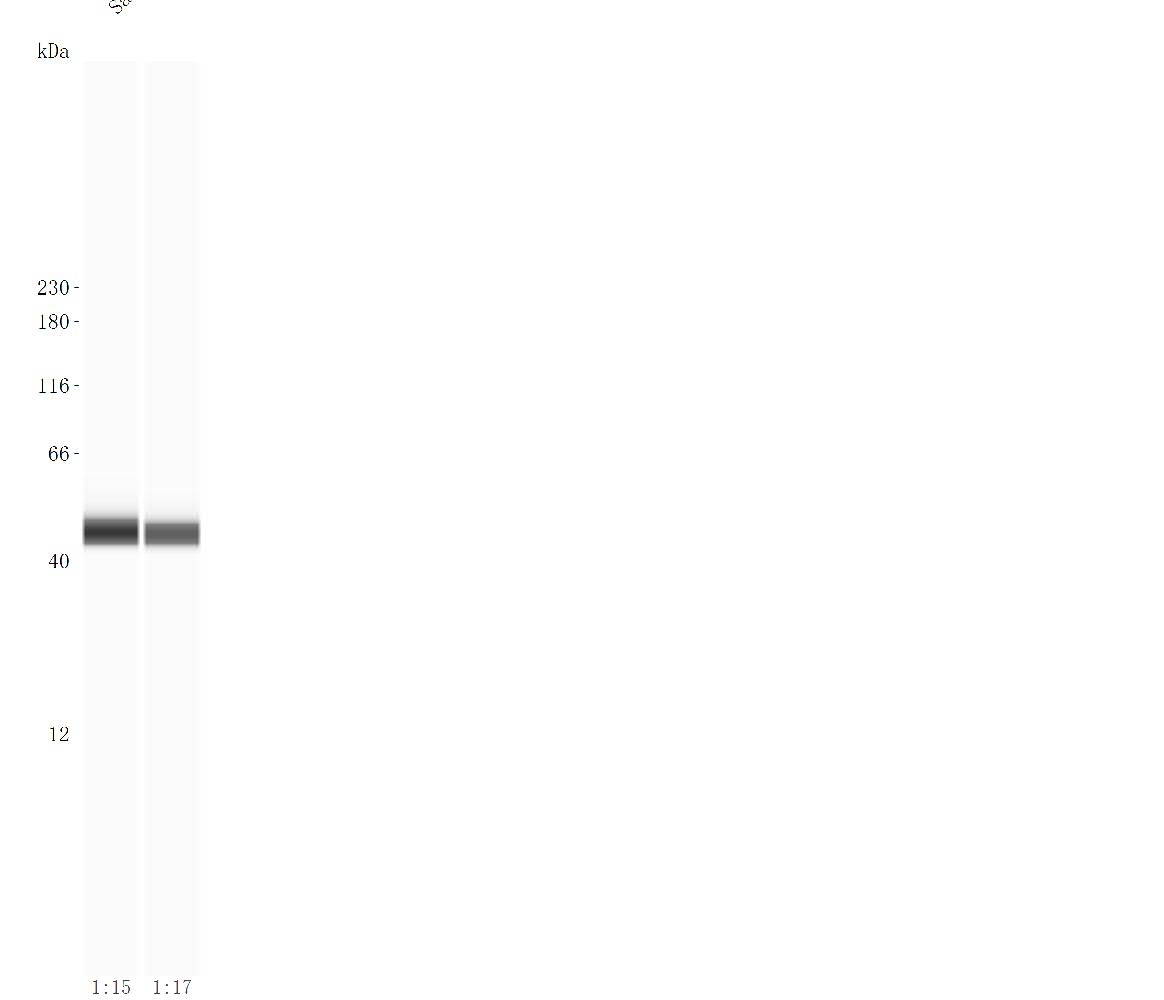

Supplement: Figure 2—source data 2. [file elife-100730-fig2-data2.zip › Figure 2L OCT4.jpg]

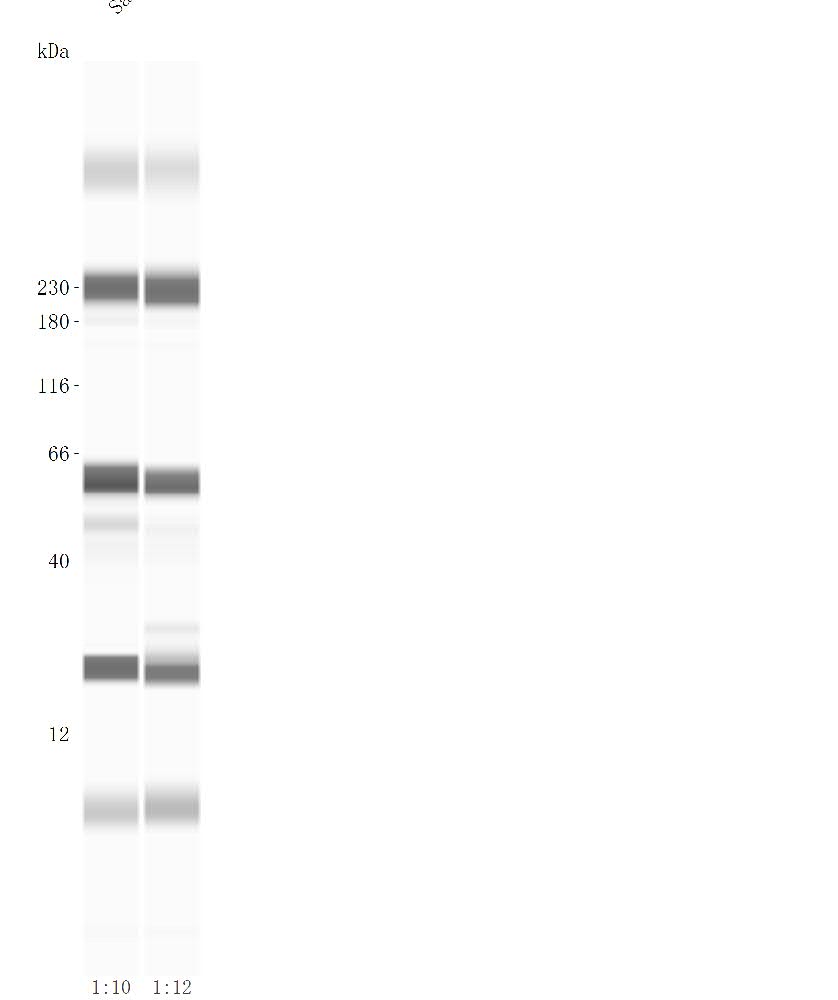

Supplement: Figure 2—source data 2. [file elife-100730-fig2-data2.zip › Figure 2L SOX2.jpg]

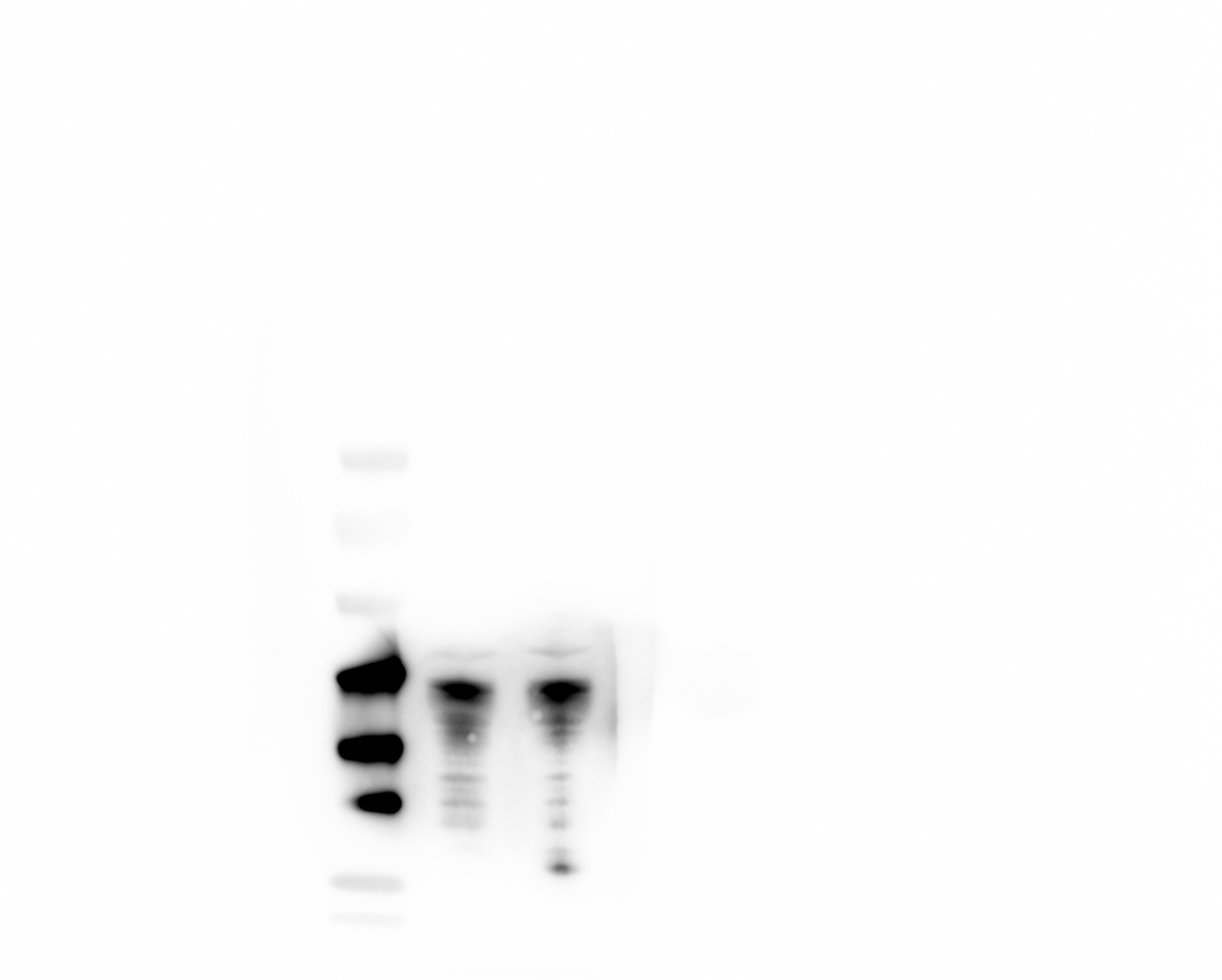

Supplement: Figure 2—source data 2. [file elife-100730-fig2-data2.zip › Figure 2M CDX2.Tif]

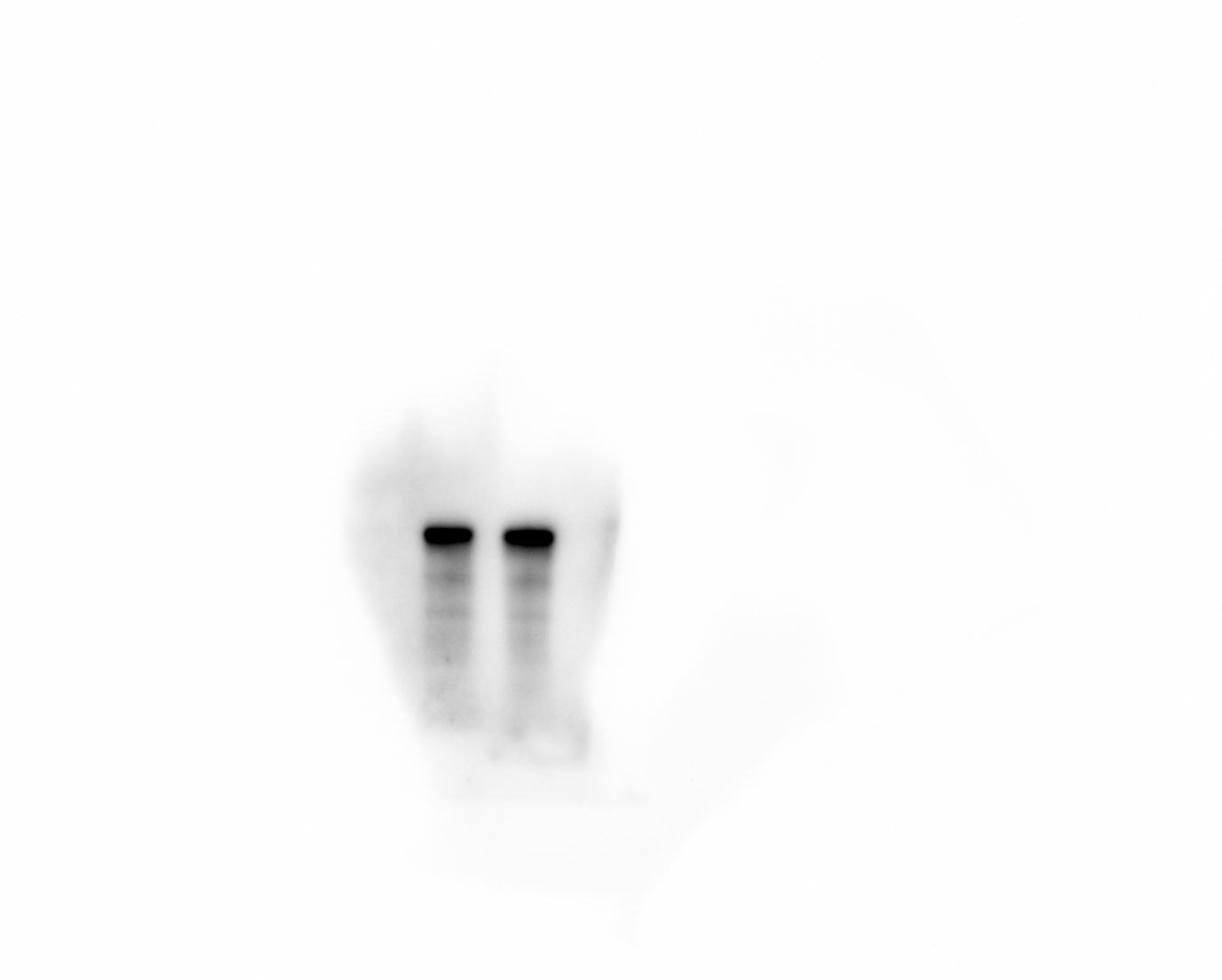

Supplement: Figure 2—source data 2. [file elife-100730-fig2-data2.zip › Figure 2M GAPDH.Tif]

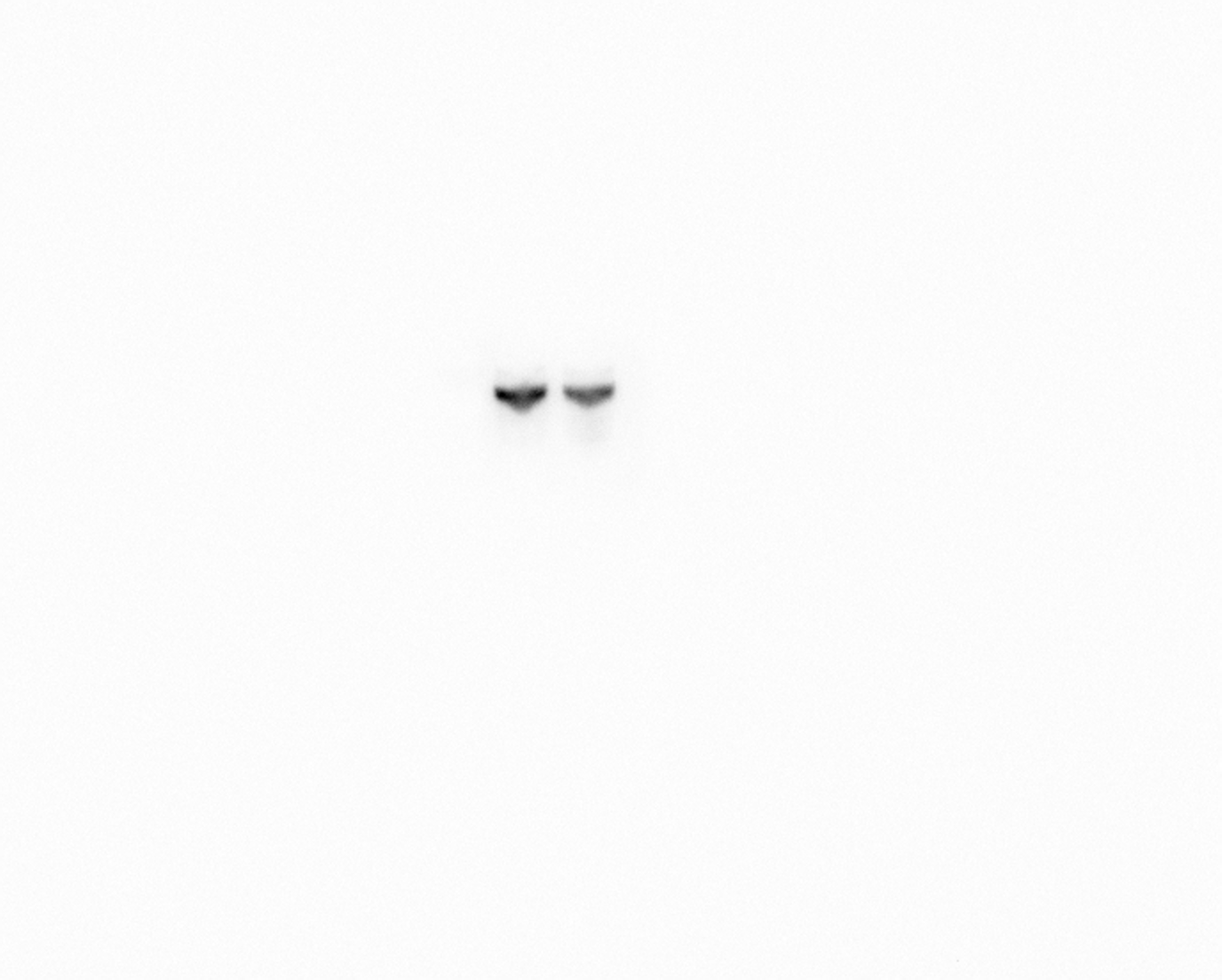

Supplement: Figure 2—source data 2. [file elife-100730-fig2-data2.zip › Figure 2M HSPA2.Tif]

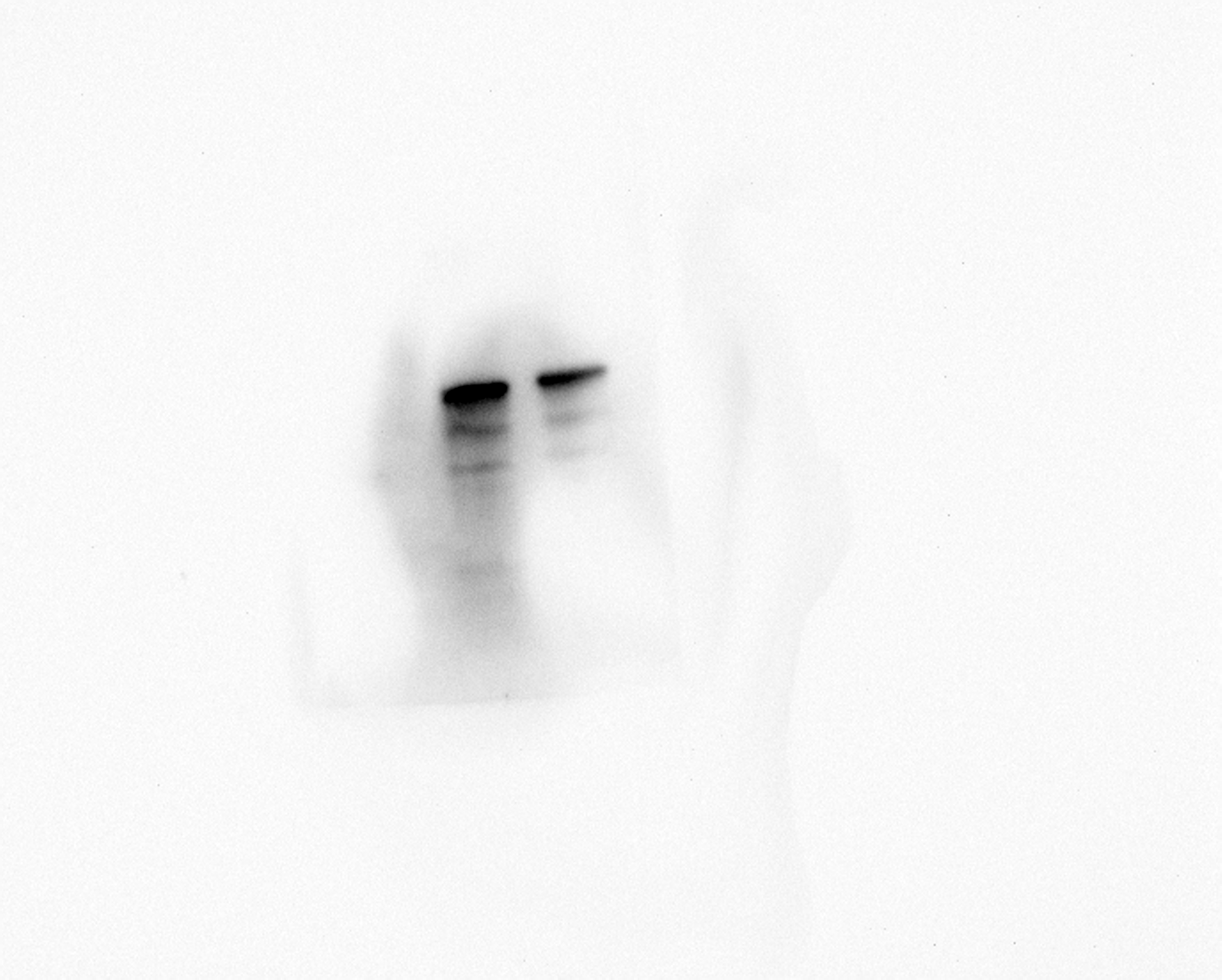

Supplement: Figure 2—source data 2. [file elife-100730-fig2-data2.zip › Figure 2M NANOG.Tif]

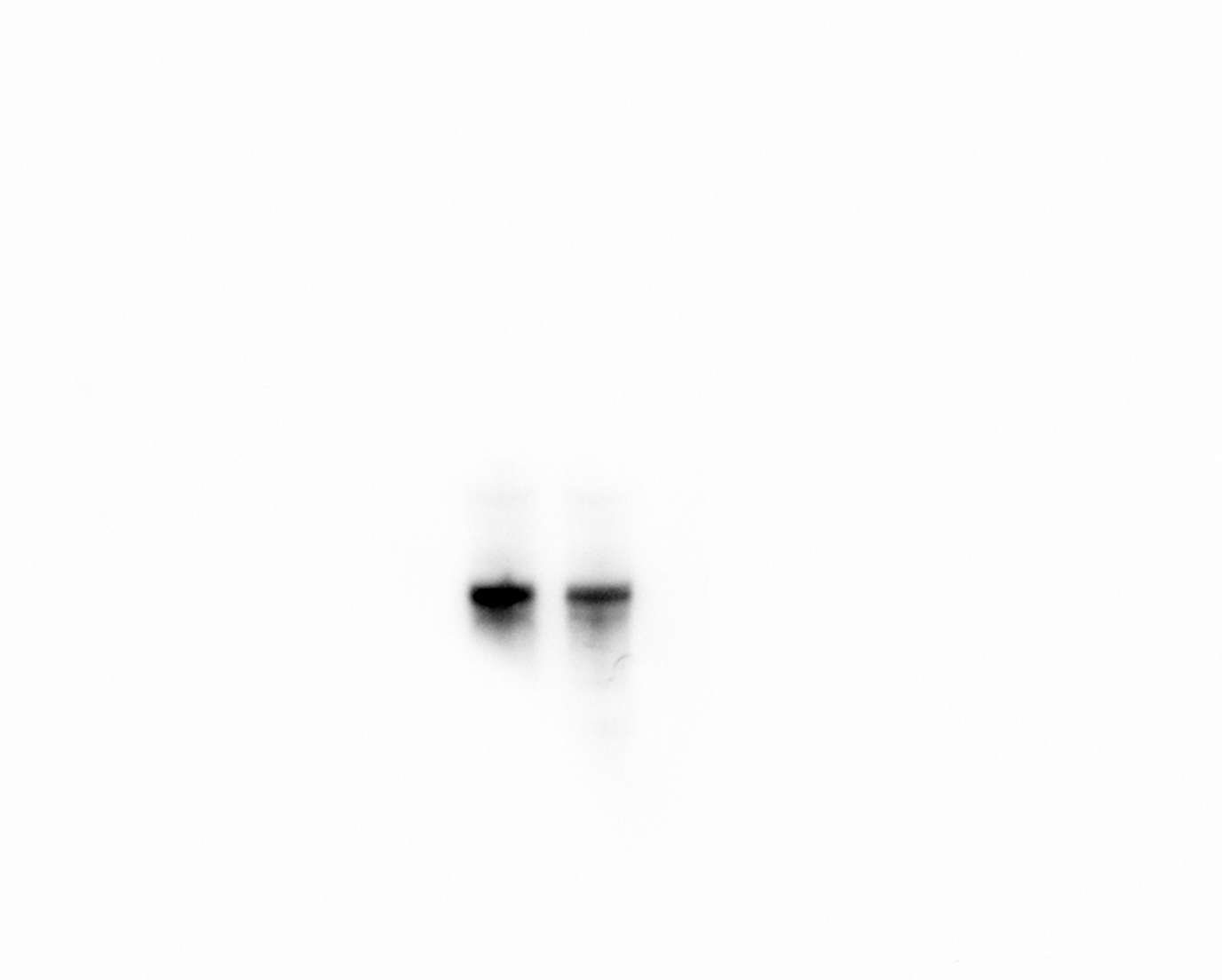

Supplement: Figure 2—source data 2. [file elife-100730-fig2-data2.zip › Figure 2M OCT4.Tif]

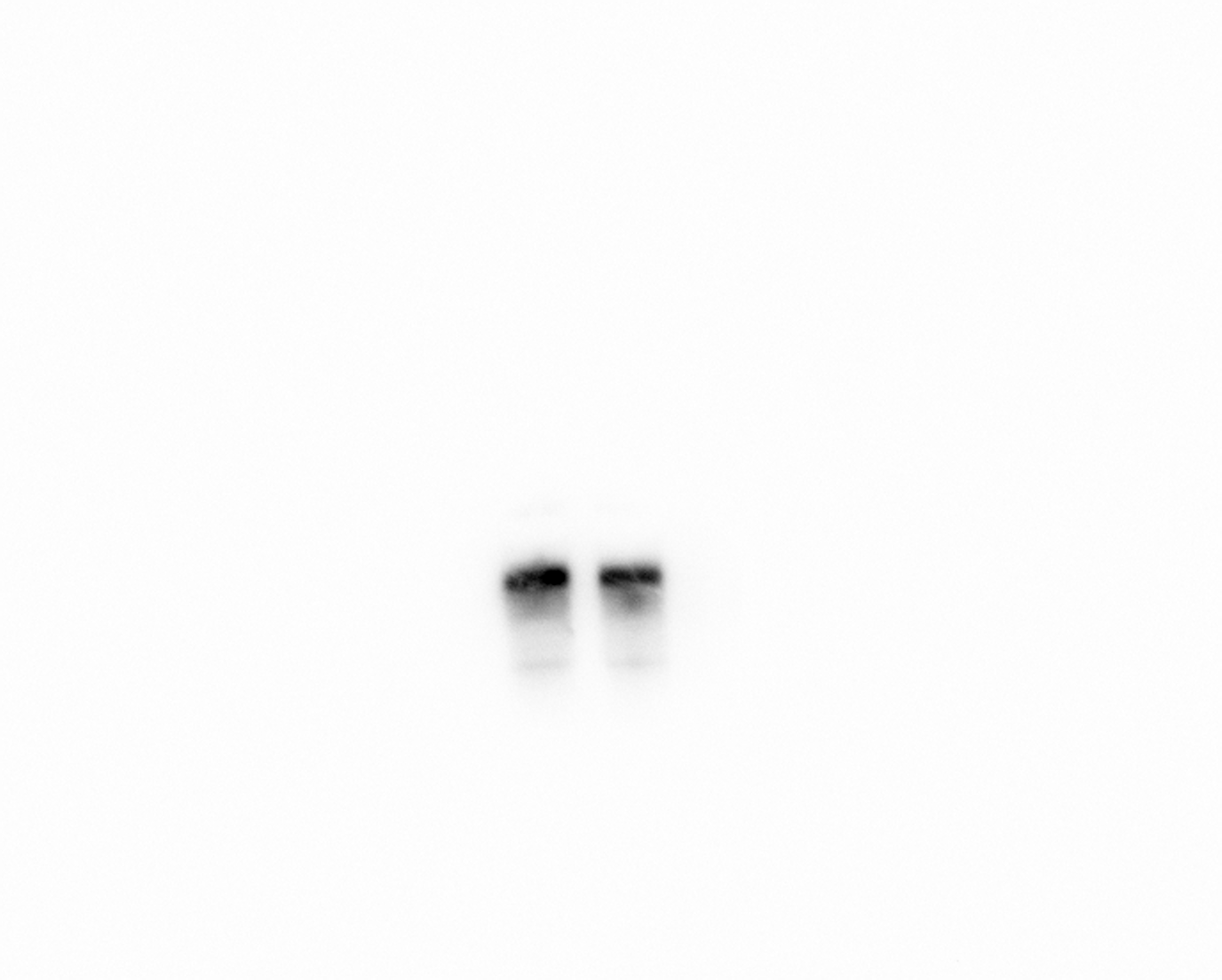

Supplement: Figure 2—source data 2. [file elife-100730-fig2-data2.zip › Figure 2M SOX2.Tif]

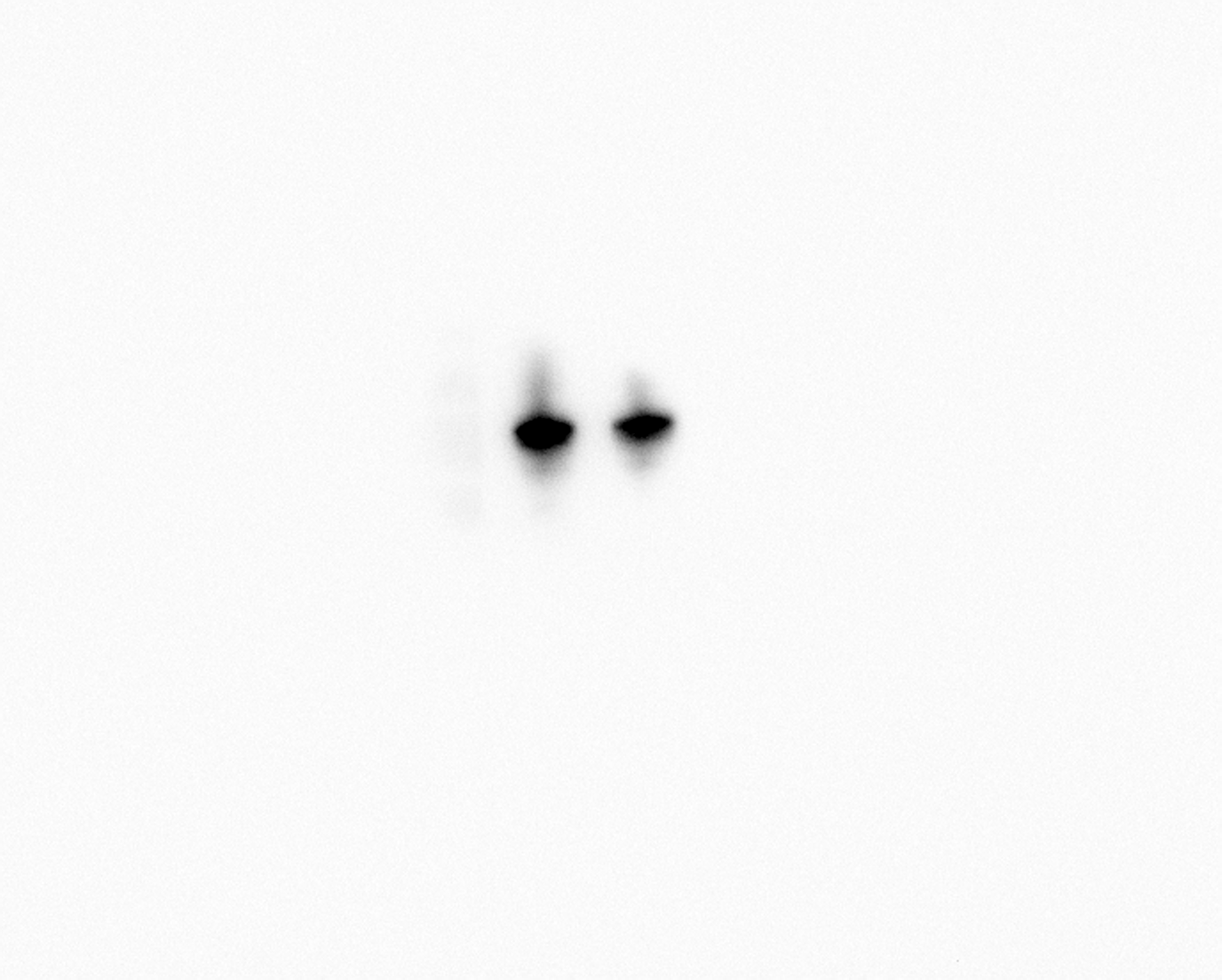

Supplement: Figure 4—figure supplement 1—source data 2. [file elife-100730-fig4-figsupp1-data2.zip › GAPDH.Tif]

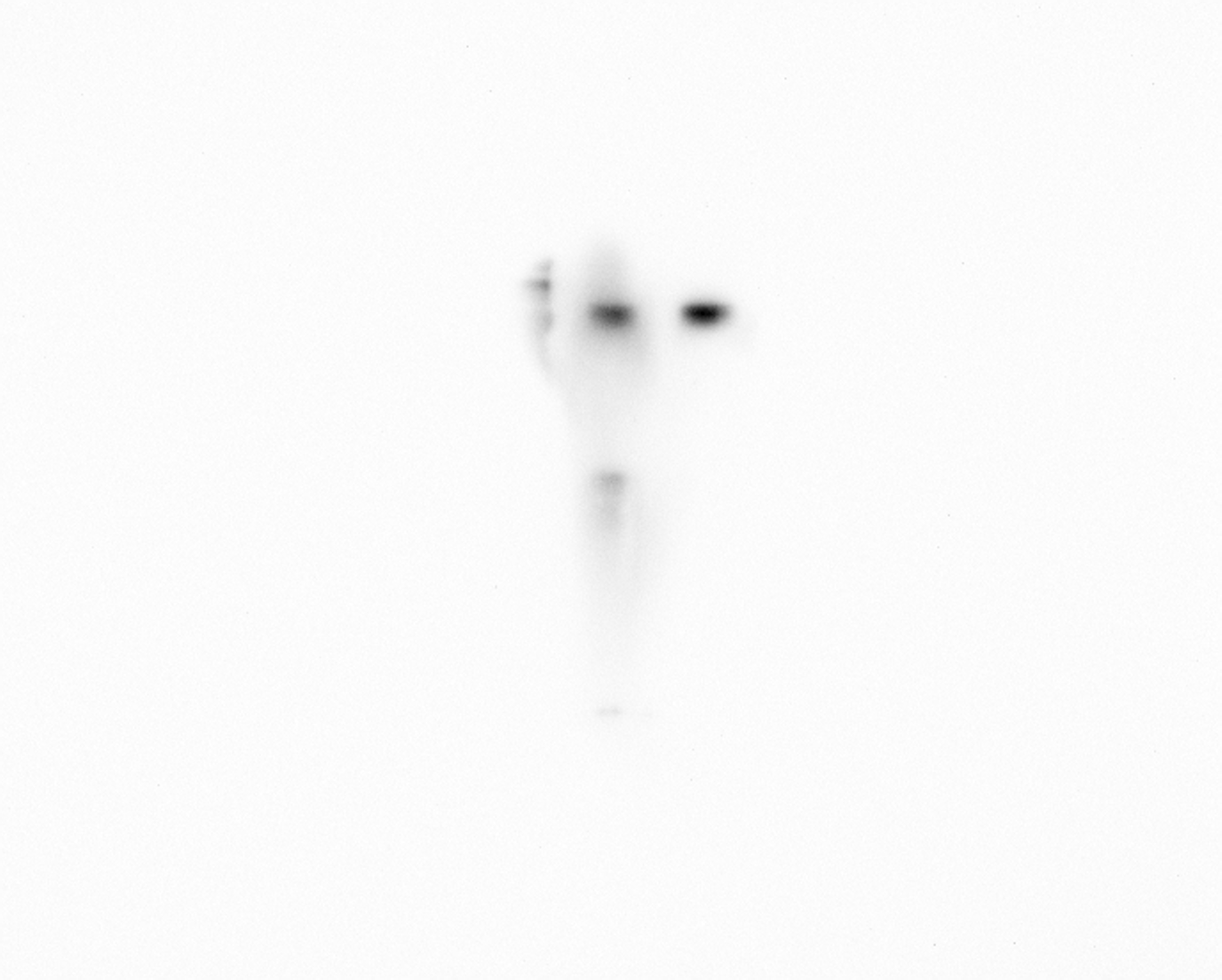

Supplement: Figure 4—figure supplement 1—source data 2. [file elife-100730-fig4-figsupp1-data2.zip › HSPA2.Tif]

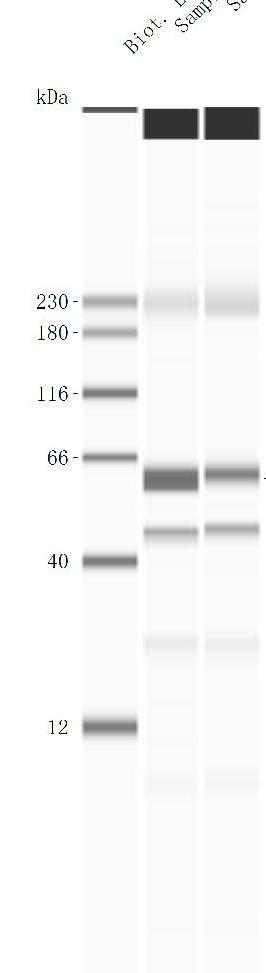

Supplement: Figure 5—source data 2. [file elife-100730-fig5-data2.zip › Figure 5D CARM1.jpg]

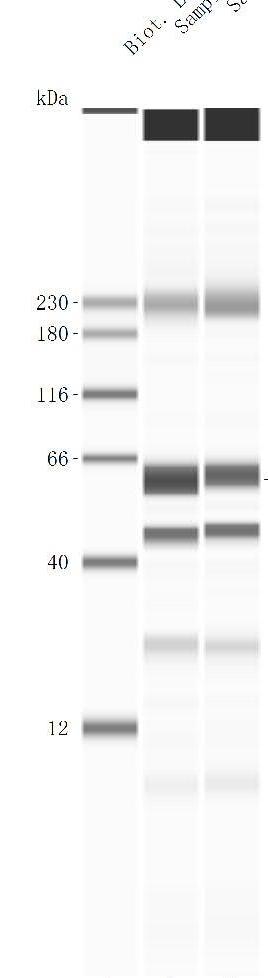

Supplement: Figure 5—source data 2. [file elife-100730-fig5-data2.zip › Figure 5D GAPDH.jpg]

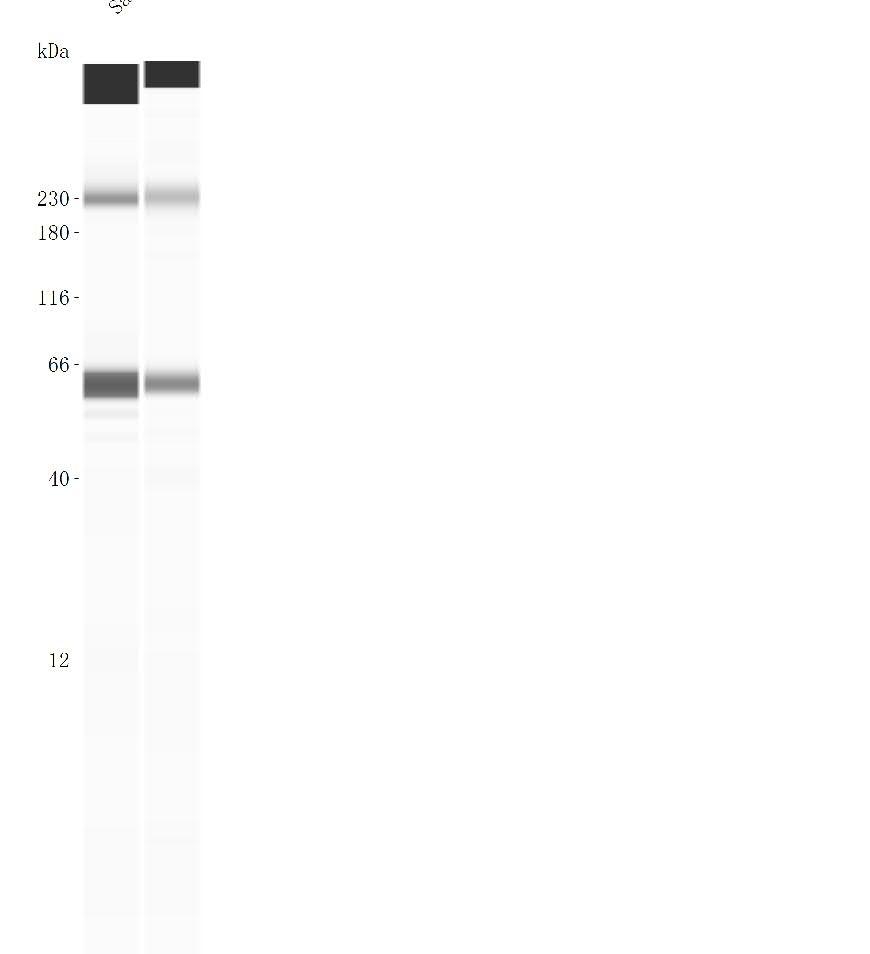

Supplement: Figure 5—source data 2. [file elife-100730-fig5-data2.zip › Figure 5E CARM1.jpg]

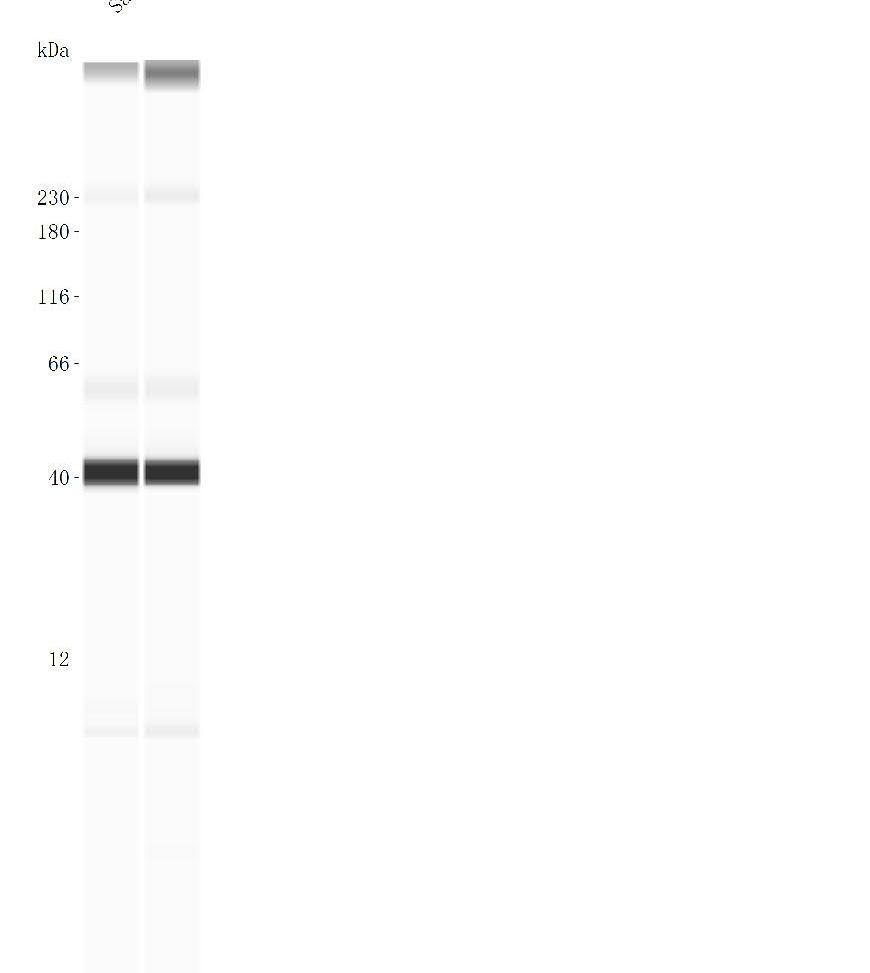

Supplement: Figure 5—source data 2. [file elife-100730-fig5-data2.zip › Figure 5E GAPDH.jpg]

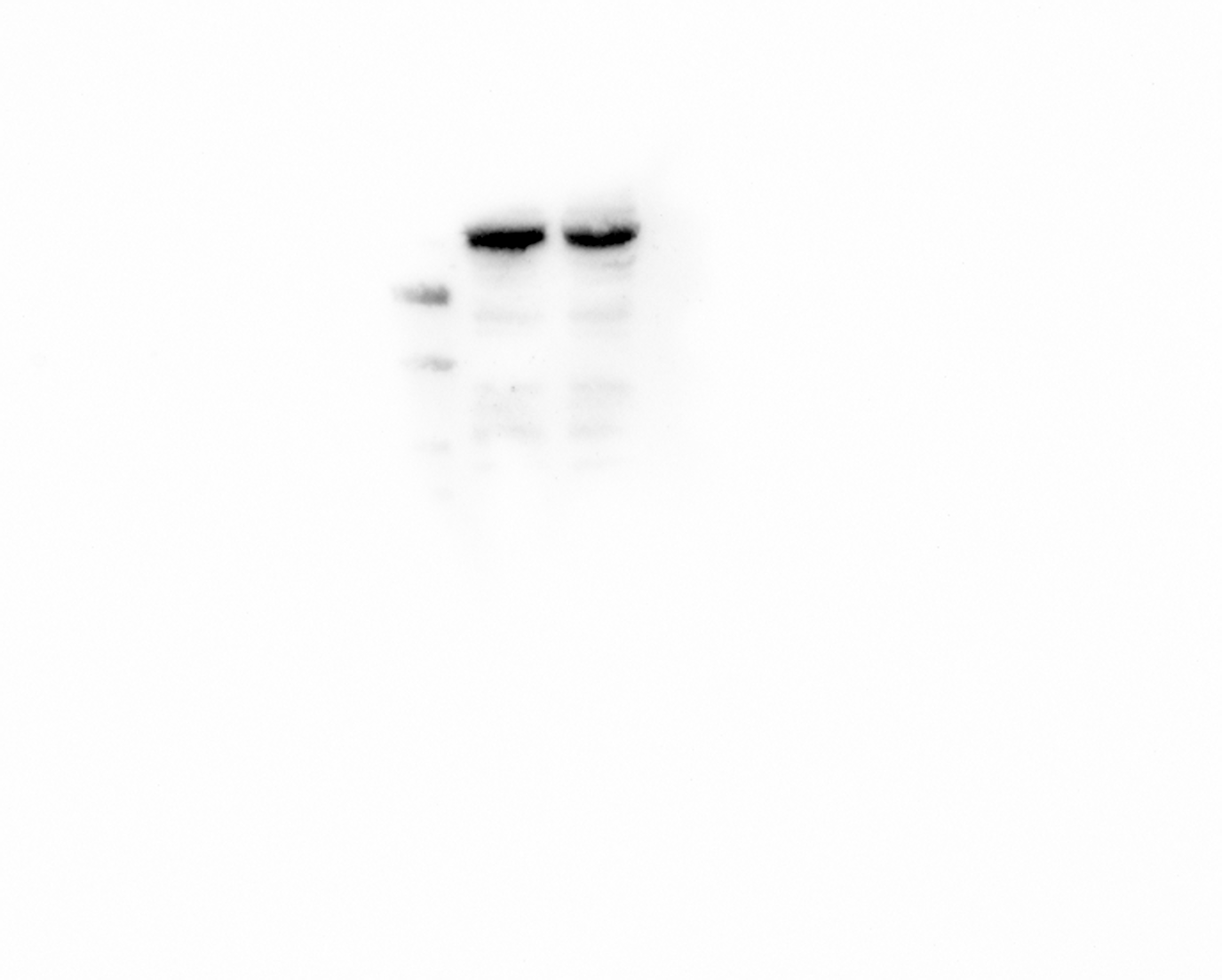

Supplement: Figure 5—source data 2. [file elife-100730-fig5-data2.zip › Figure 5F input CARM1.Tif]

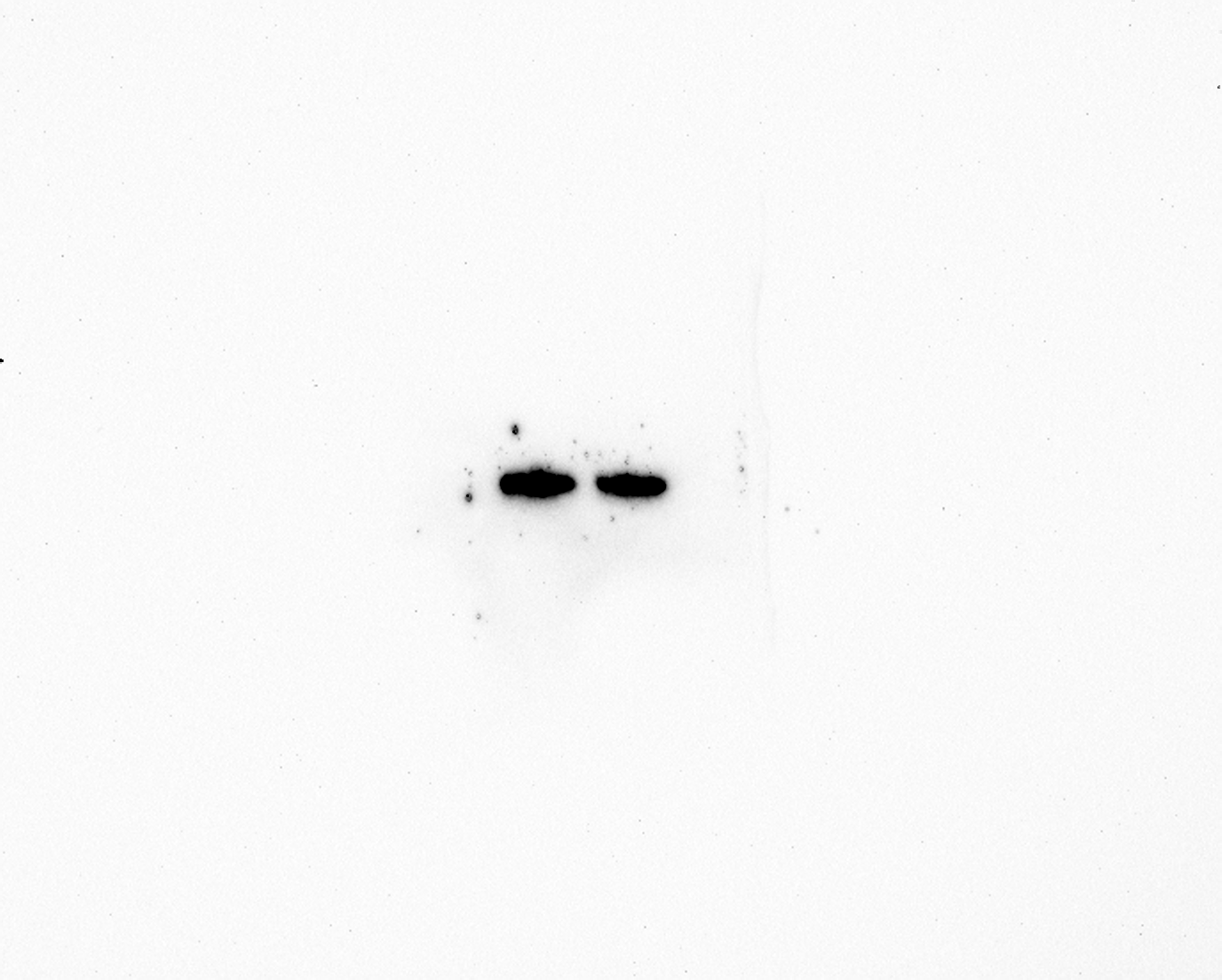

Supplement: Figure 5—source data 2. [file elife-100730-fig5-data2.zip › Figure 5F input GAPDH.Tif]

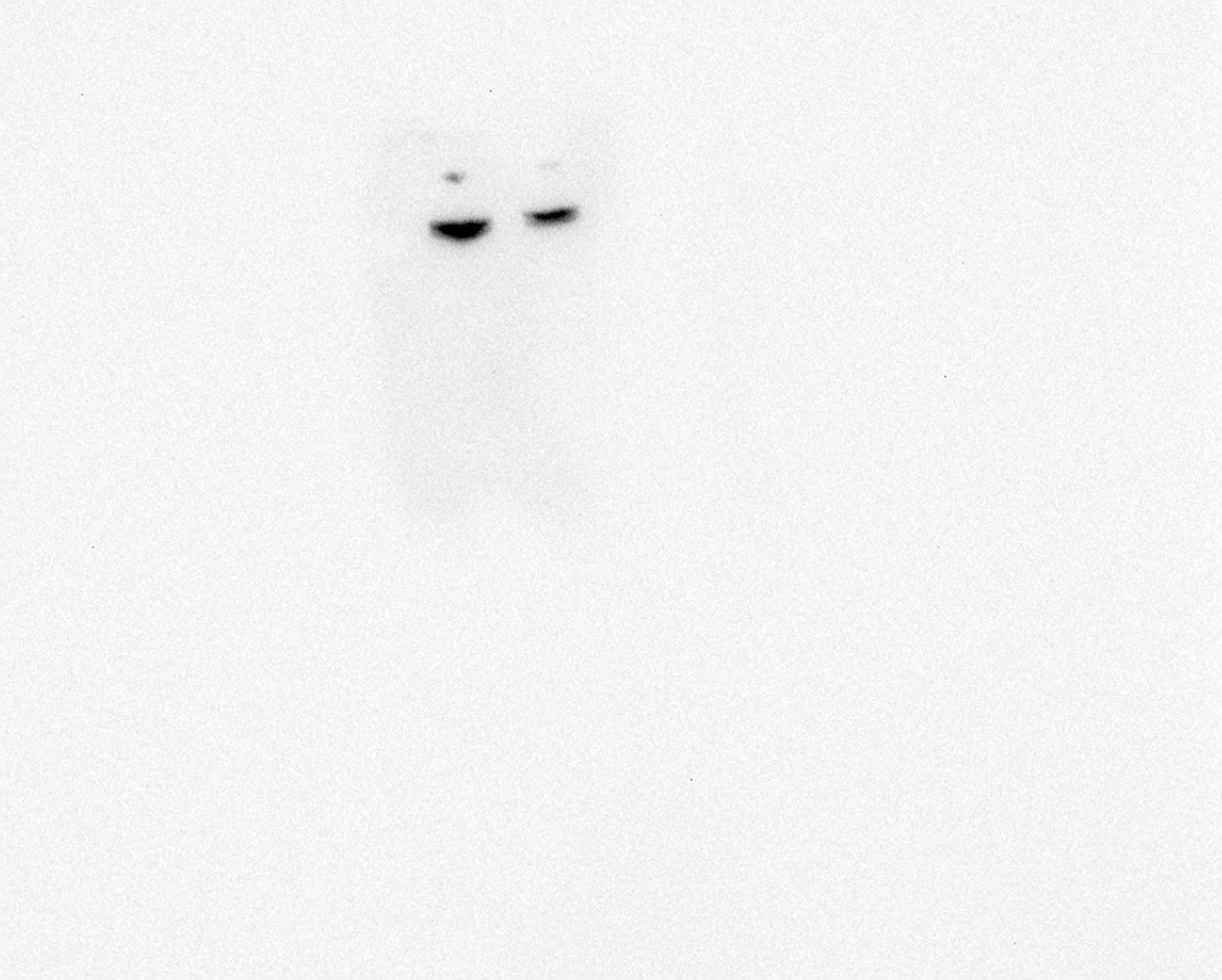

Supplement: Figure 5—source data 2. [file elife-100730-fig5-data2.zip › Figure 5F input HSPA2.Tif]

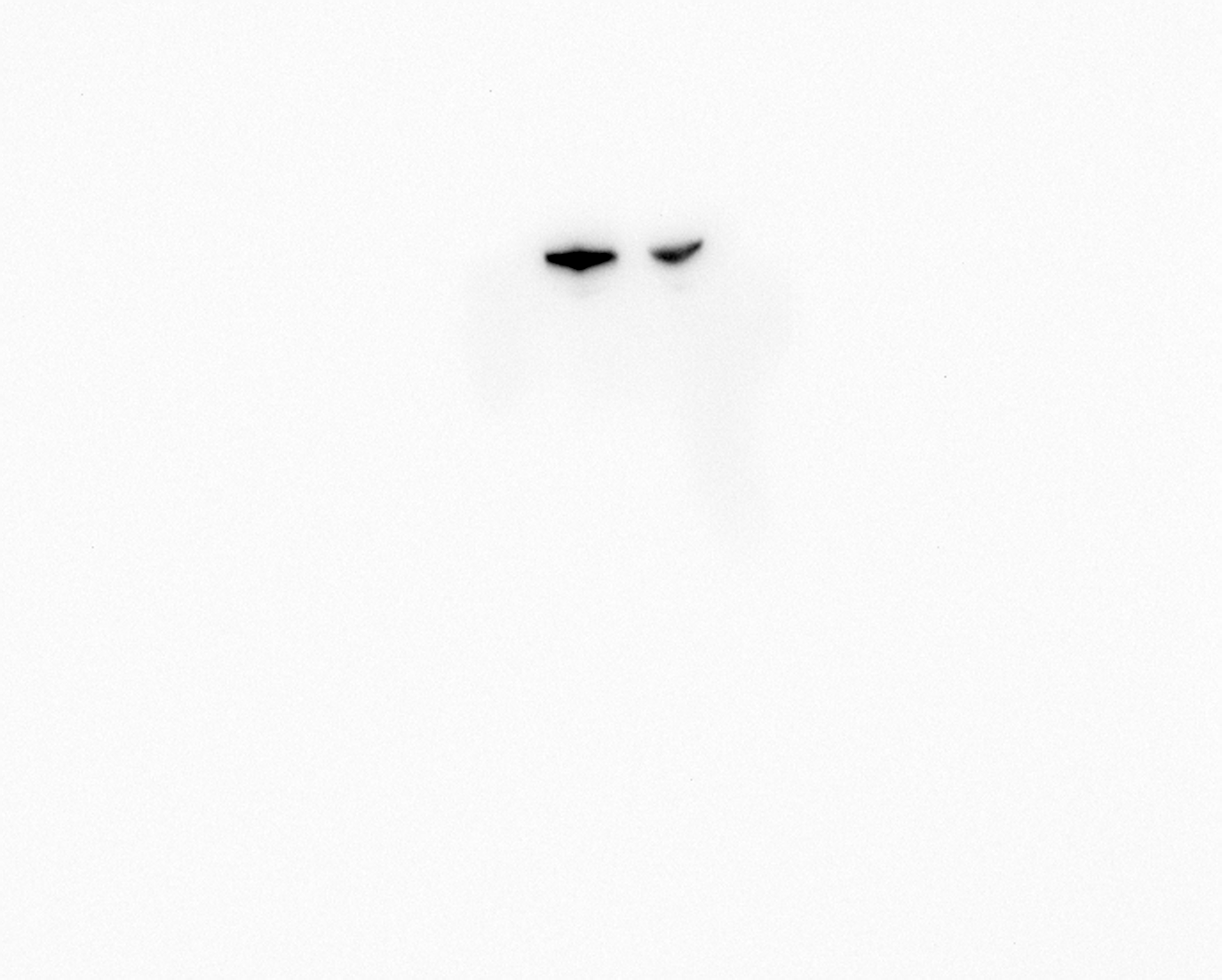

Supplement: Figure 5—source data 2. [file elife-100730-fig5-data2.zip › Figure 5F ip CARM1.Tif]

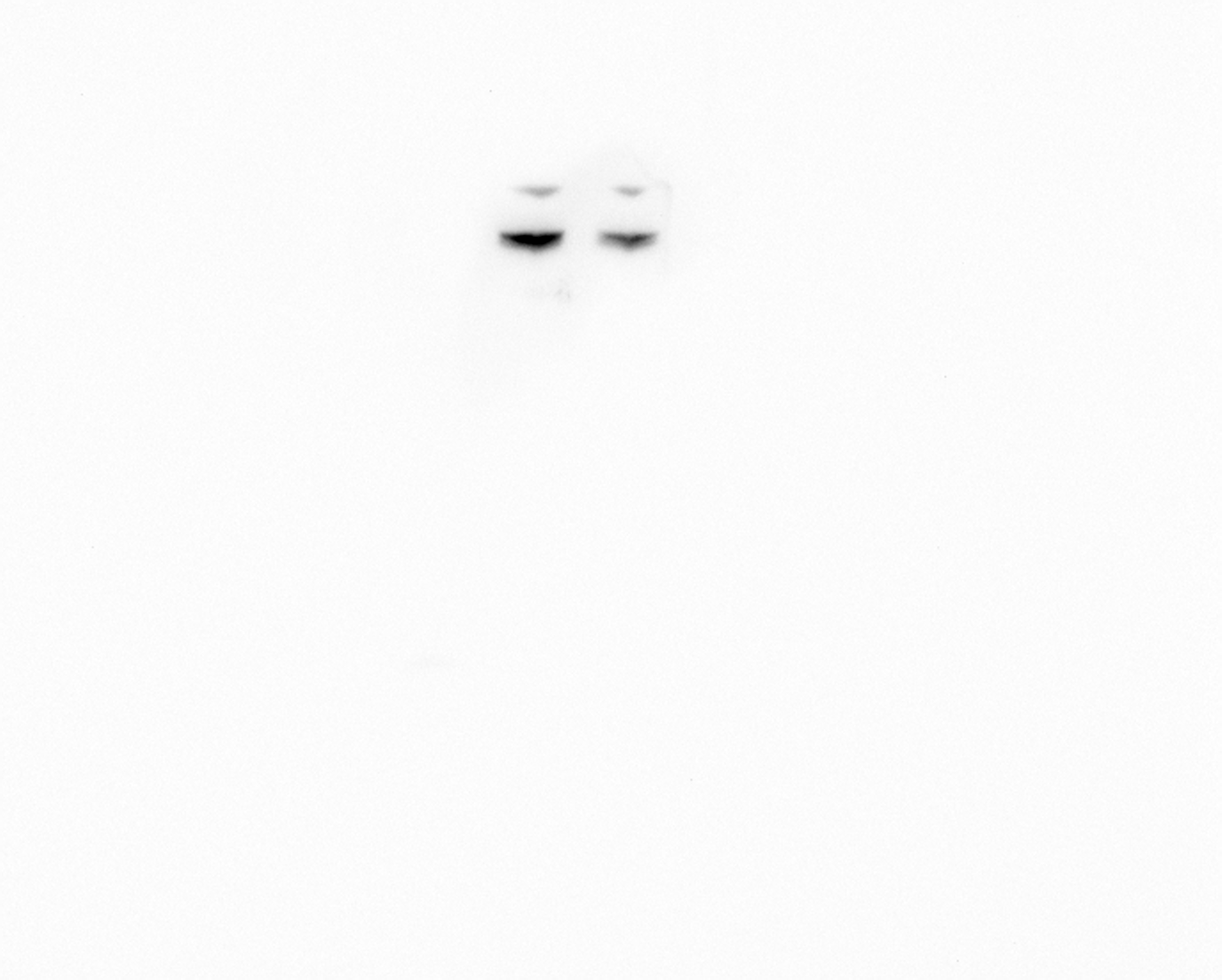

Supplement: Figure 5—source data 2. [file elife-100730-fig5-data2.zip › Figure 5F ip HSPA2.Tif]

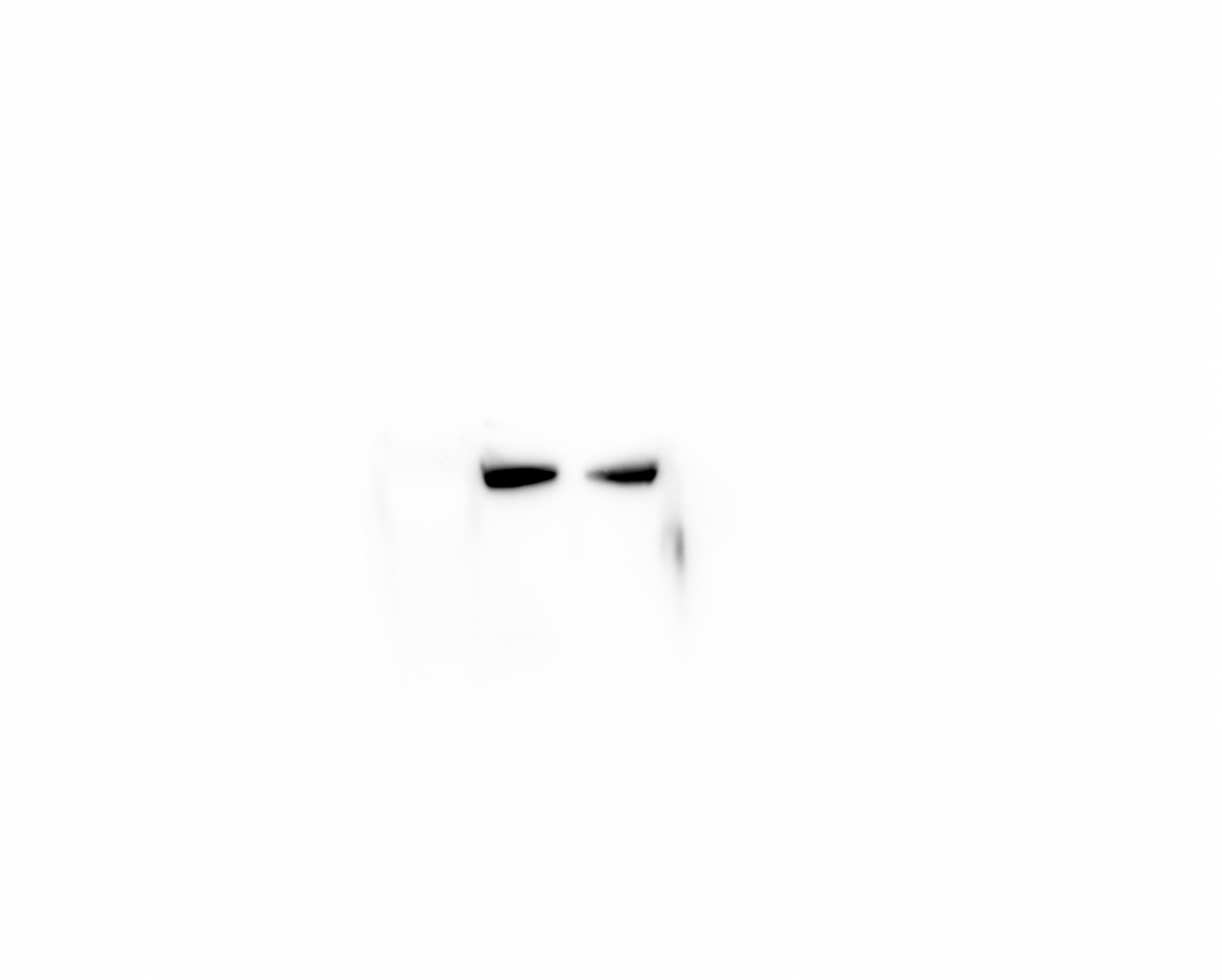

Supplement: Figure 5—source data 2. [file elife-100730-fig5-data2.zip › Figure 5G input HSPA2-.Tif]

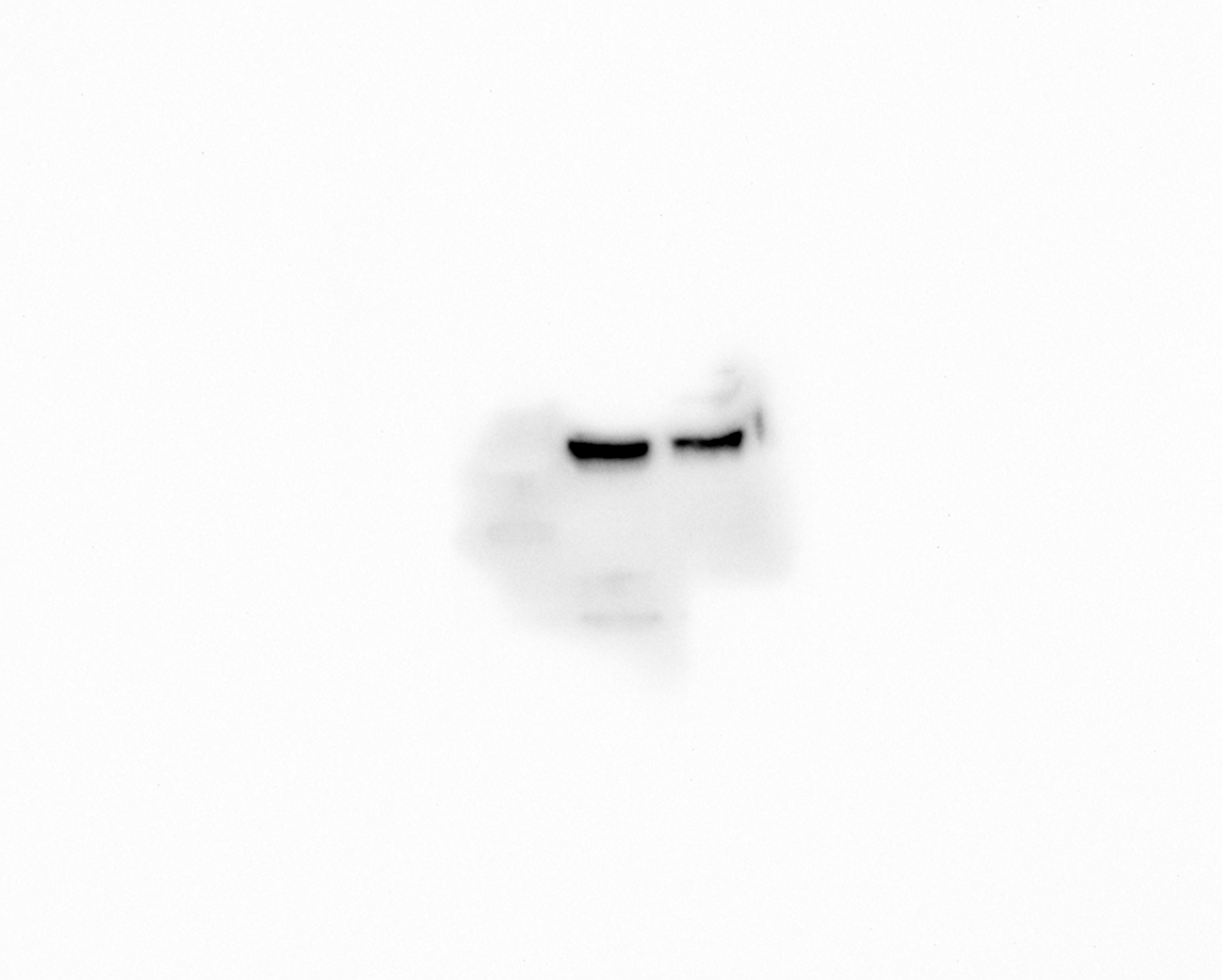

Supplement: Figure 5—source data 2. [file elife-100730-fig5-data2.zip › Figure 5G input CARM1.Tif]

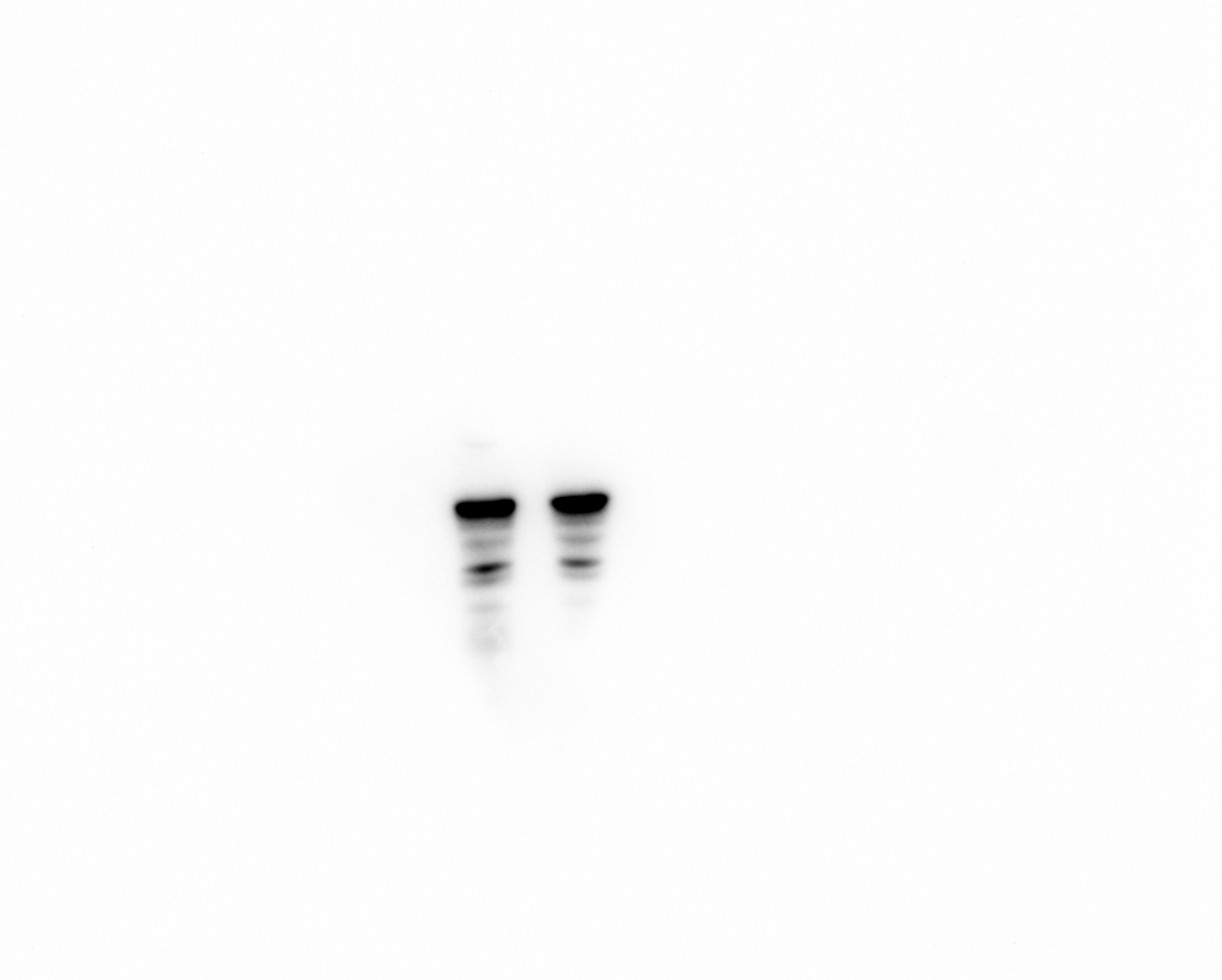

Supplement: Figure 5—source data 2. [file elife-100730-fig5-data2.zip › Figure 5G input GAPDH.Tif]

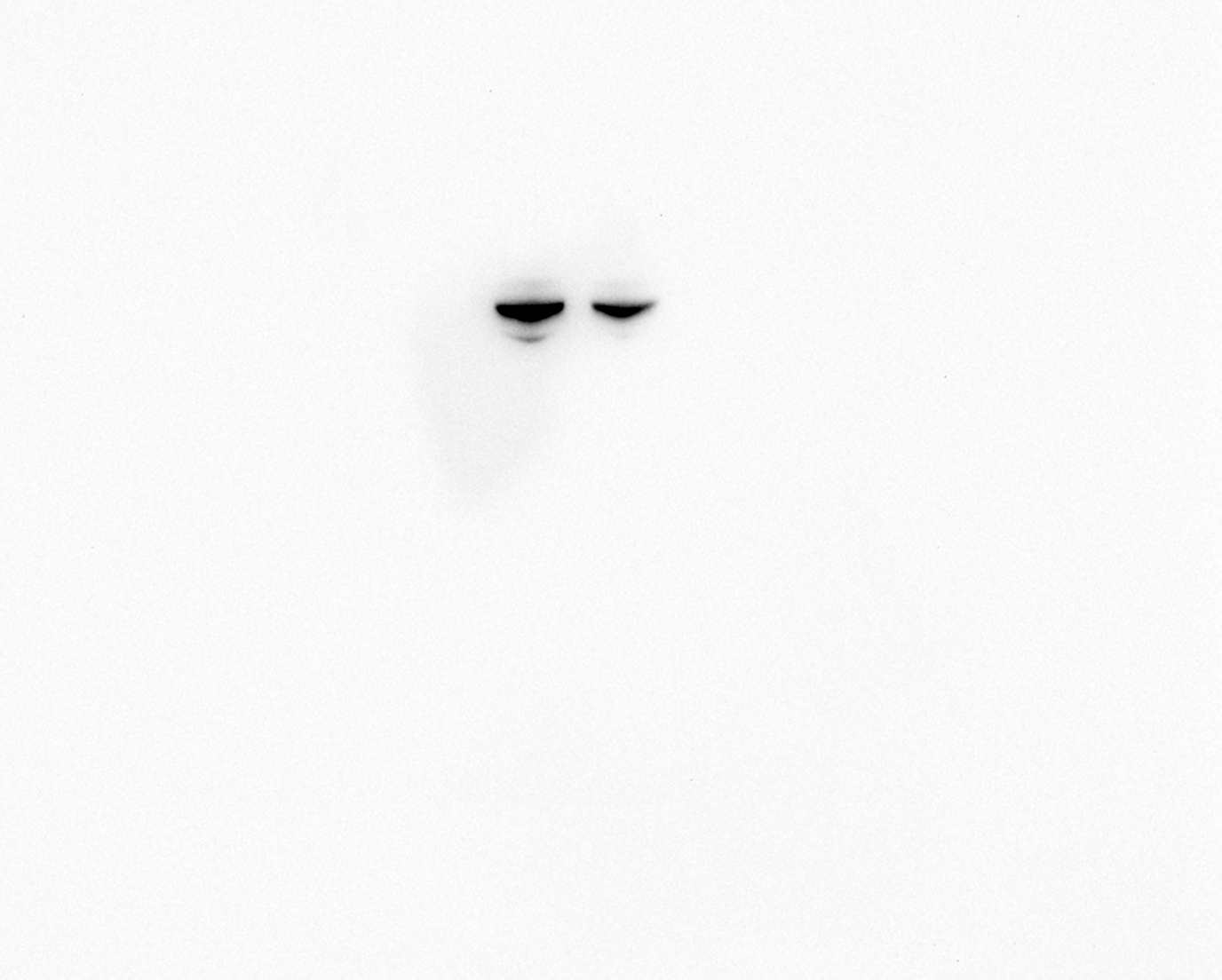

Supplement: Figure 5—source data 2. [file elife-100730-fig5-data2.zip › Figure 5G ip CARM1.Tif]

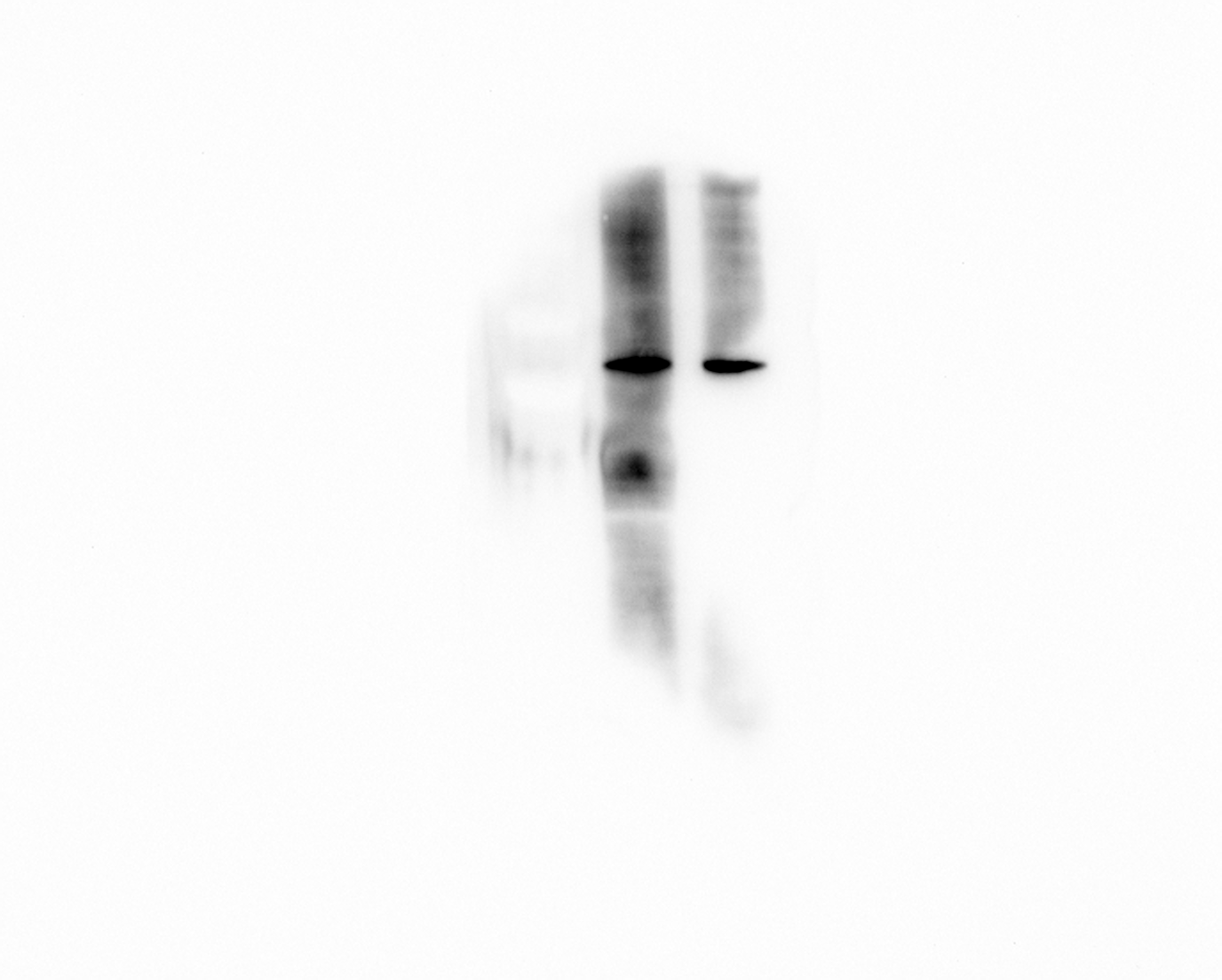

Supplement: Figure 5—source data 2. [file elife-100730-fig5-data2.zip › Figure 5G ip HASPA2.Tif]

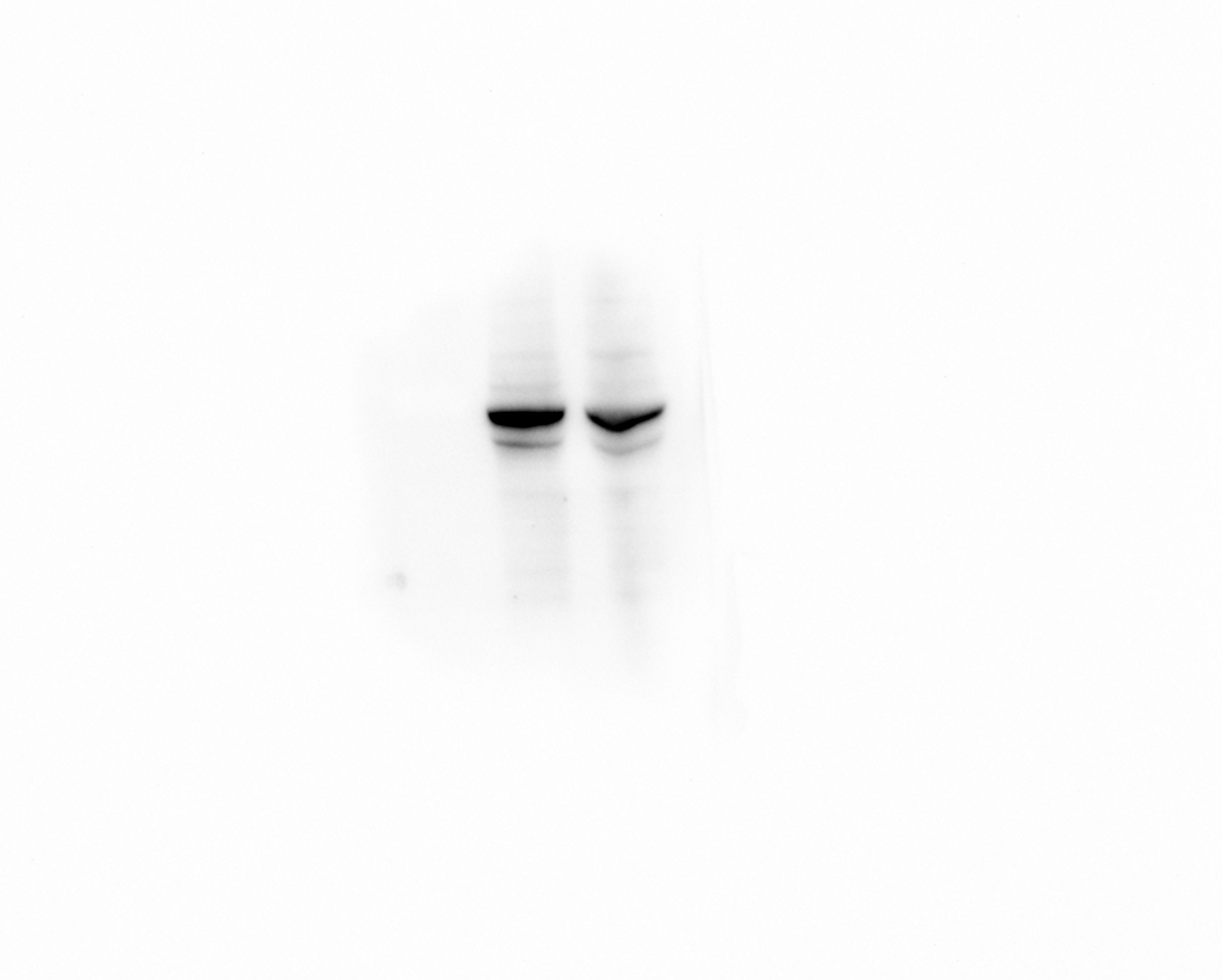

Supplement: Figure 5—figure supplement 1—source data 2. [file elife-100730-fig5-figsupp1-data2.zip › CARM1.Tif]

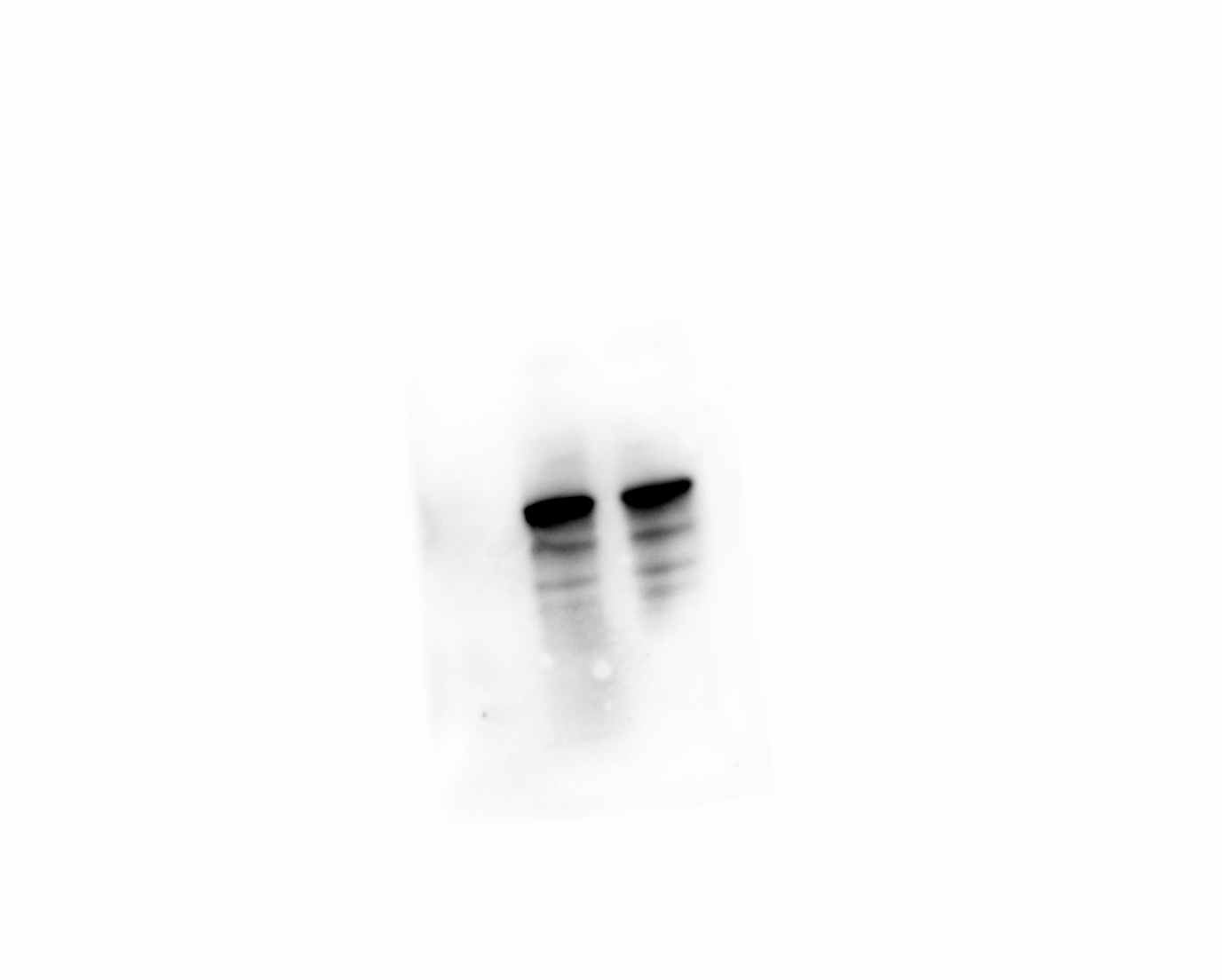

Supplement: Figure 5—figure supplement 1—source data 2. [file elife-100730-fig5-figsupp1-data2.zip › GAPDH.Tif]
