# Supplementary material for: The asymmetric expression of HSPA2 in blastomeres governs the first embryonic cell-fate decision
Source: eLife. 2025 Mar 10;13:RP100730. doi: 10.7554/eLife.100730 (PMC11893103; doi:10.7554/eLife.100730)
Supplement: Figure 1—figure supplement 1—source data 1. [file elife-100730-fig1-figsupp1-data1.zip › Figure 1—figure supplement 1—source data 1.pdf]

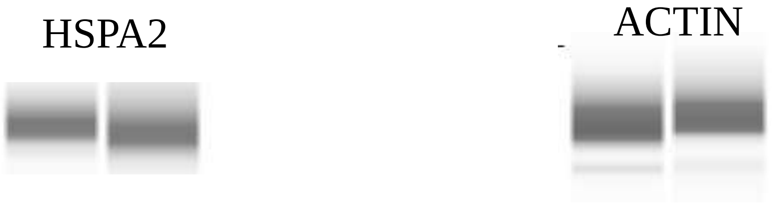

Figure 1—figure supplement 1—source data 1. Original membranes corresponding to Figure 1—figure supplement 1, panel D.
