# Supplementary material for: The asymmetric expression of HSPA2 in blastomeres governs the first embryonic cell-fate decision
Source: eLife. 2025 Mar 10;13:RP100730. doi: 10.7554/eLife.100730 (PMC11893103; doi:10.7554/eLife.100730)
Supplement: Figure 5—source data 1. [file elife-100730-fig5-data1.zip › Figure 5–Source Data 1.pdf]

CARM1

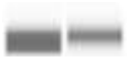

GAPDH

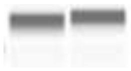

Figure 5, Source Data 1. Original membranes corresponding to Figure 5, panel D.

CARM1

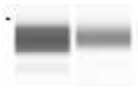

GAPDH

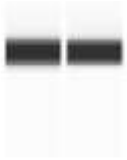

Figure 5, Source Data 1. Original membranes corresponding to Figure 5, panel E.

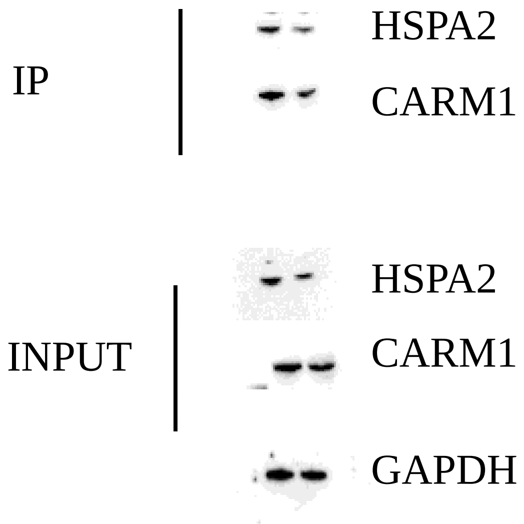

Figure 5, Source Data 1. Original membranes corresponding to Figure 5, panel F.

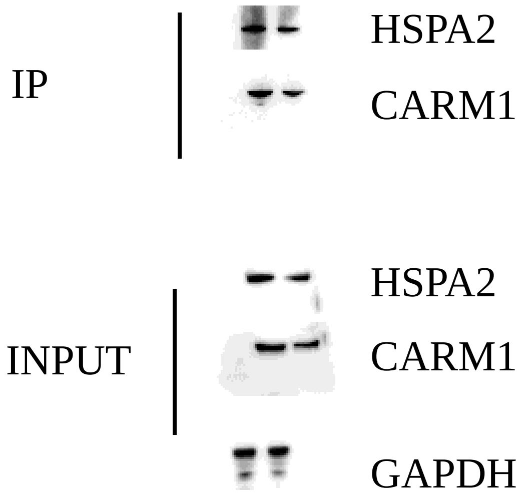

Figure 5, Source Data 1. Original membranes corresponding to Figure 5, panel G.
